# Effectiveness of psychosocial interventions in eating disorders: an overview of Cochrane systematic reviews

**DOI:** 10.1590/S1679-45082016RW3120

**Published:** 2016

**Authors:** Marcelle Barrueco Costa, Tamara Melnik

**Affiliations:** 1 Universidade Federal de São Paulo, São Paulo, SP, Brazil;; Centro Cochrane do Brasil, São Paulo, SP, Brazil.

**Keywords:** Eating disorders, Evidence-based medicine

## Abstract

Eating disorders are psychiatric conditions originated from and perpetuated by individual, family and sociocultural factors. The psychosocial approach to treatment and prevention of relapse is crucial. To present an overview of the scientific evidence on effectiveness of psychosocial interventions in treatment of eating disorders. All systematic reviews published by the Cochrane Database of Systematic Reviews - Cochrane Library on the topic were included. Afterwards, as from the least recent date of these reviews (2001), an additional search was conducted at PubMed with sensitive search strategy and with the same keywords used. A total of 101 primary studies and 30 systematic reviews (5 Cochrane systematic reviews), meta-analysis, guidelines or narrative reviews of literature were included. The main outcomes were: symptomatic remission, body image, cognitive distortion, psychiatric comorbidity, psychosocial functioning and patient satisfaction. The cognitive behavioral approach was the most effective treatment, especially for bulimia nervosa, binge eating disorder and the night eating syndrome. For anorexia nervosa, the family approach showed greater effectiveness. Other effective approaches were interpersonal psychotherapy, dialectic behavioral therapy, support therapy and self-help manuals. Moreover, there was an increasing number of preventive and promotional approaches that addressed individual, family and social risk factors, being promising for the development of positive self-image and self-efficacy. Further studies are required to evaluate the impact of multidisciplinary approaches on all eating disorders, as well as the cost-effectiveness of some effective modalities, such as the cognitive behavioral therapy.

## RESUMO

Transtornos alimentares são doenças psiquiátricas originadas de e perpetuadas por fatores individuais, familiares e socioculturais. A abordagem psicossocial é essencial para o tratamento e a prevenção de recaídas. Apresentar uma visão geral das evidências científicas sobre a efetividade das intervenções psicossociais no tratamento de transtornos alimentares. Foram incluídas todas as revisões sistemáticas publicadas no Banco de Dados de Revisões Sistemáticas da *Cochrane Library*. Posteriormente, a partir da data menos recente destas revisões (2001), realizou-se uma busca adicional no PubMed, com estratégia de busca sensibilizada e com os mesmos descritores utilizados antes. No total, foram incluídos 101 estudos primários e 30 revisões sistemáticas (5 revisões sistemáticas da Cochrane), metanálises, diretrizes ou revisões narrativas da literatura. Os principais desfechos foram remissão de sintomas, imagem corporal, distorção cognitiva, comorbidade psiquiátrica, funcionamento psicossocial e satisfação do paciente. A abordagem cognitivo-comportamental foi o tratamento mais efetivo, principalmente para bulimia nervosa, transtorno da compulsão alimentar periódica e síndrome do comer noturno. Para anorexia nervosa, a abordagem familiar demonstrou maior efetividade. Outras abordagens efetivas foram psicoterapia interpessoal, terapia comportamental dialética, terapia de apoio e manuais de autoajuda. Além disso, houve um número crescente de abordagens preventivas e promocionais que contemplaram fatores de risco individuais, familiares e sociais, sendo promissoras para o desenvolvimento da autoimagem positiva e autoeficácia. São necessários mais estudos que avaliem o impacto de abordagens multidisciplinares em todos transtornos alimentares, além da relação custo-efetividade de algumas modalidades efetivas, como a terapia cognitivo-comportamental.

## INTRODUCTION

The eating disorders have specific diagnoses, including anorexia nervosa (AN), bulimia nervosa (BN) and binge eating disorder (BED).^(1)^ However, subclinical forms are more frequent across all age groups.^(2-4)^ Along with subclinical forms, BED is more common than AN and BN, and due to its specific clinical manifestation and strong association with obesity, it is classified as a diagnostic category in fifth edition of the Diagnostic and Statistical Manual of Mental Disorders (DSM-V).^(1-3,5)^ The prevalence of BED in the United States within the period of 12 months is 1.6% for women and 0.8% for men.^(1)^ The prevalence between genders in BED is more similar than AN or BN, which predominate in young females.^(1)^ The 12-month prevalence of AN in these women is approximately 0.4%, and of BN varies from 1 to 1.5%.^(1)^

AN is characterized by restriction of energy intake relative to requirements, leading to a significantly low body weight; intense fear of gaining weight or of becoming fat, or persistent behavior that interferes with weight gain; disturbance in the way in which one’s body weight or shape is experienced, undue influence of body weight or shape on self-evaluation, or persistent lack of recognition of the seriousness of the current low body weight.^(1)^ BN is characterized by repetitive episodes of binge eating (eating an amount of food that is definitely larger than what most individuals would eat in a similar period of time under similar circumstances with a sense of lack of control), followed by compensatory behaviors (vomiting, laxative use, and excessive physical activity) in an attempt to undo the excessive intake of food, as well as a disturbance in the perception of shape and weight, like in AN.^(1)^ These episodes occur at least twice a week for a minimum period of 3 months.^(1)^ In BED, the same episodes occur with similar frequency and duration, but patients do not generally have regular compensatory behaviors to combat excessive consumption of food and often present with overweight or obesity.^(1)^

The other specified eating disorder category includes other eating symptoms that result in clinically significant distress or impairment in social functioning, but do not meet the full criteria for the three categories mentioned above (AN, BN and BED). This category includes atypical AN (all criteria, except low body weight); BN of low frequency and/or limited duration; binge-eating disorder of low frequency and/or limited duration; purging disorder (compensatory behaviors without binge eating); night eating syndrome (NES − recurrent episodes of night eating, as manifested by eating after awakening from sleep or by excessive food consumption after the evening meal).^(1)^ The NES can be distinguished from BN and BED, mainly by the lack of compensatory behaviours associated in an attempt to counteract the excessive food intake that occurs in BN, and by the time of excessive food intake (night), unlike BED, in which binge eating episodes can occur at any time of the day.^(1)^

The psychosocial interventions are important for effective eating disorders treatments in the long run, since they address psychological and social factors involved in onset and maintenance of this disorders.^(2,4)^ The cognitive behavioral therapy (CBT), *e.g.*, was accepted as one of the main treatments for eating disorders.^(2-4)^ There is strong evidence of efficacy for bulimic symptoms (binge eating and compensatory behaviors) in BN and BED using some techniques, such as cognitive restructuring and regulation of feeding.^(2)^ The model of CBT for eating disorders is based on the fact that dysfunctional beliefs (regarding thinness and dissatisfaction with the physical shape and body weight) maintain the abnormal eating behavior and related characteristics, such as purgation and abuse of laxatives, diuretics and diet pills.^(2)^ The CBT approach is multimodal and includes nutritional counseling, psychoeducation, self-monitoring, as well as cognitive and behavioral interventions.^(2)^ The analysis of the family context is very relevant, especially in children and adolescents at risk. It is known that interventions that aimed at fast and timely improvement, focusing only on nutritional counseling or medication, are often not effective; thus, the approach of individual, family and social factors is required for both weight loss and regain to be achieved and maintained, depending on the type of eating disorders.^(2-4)^

## OBJECTIVE

The objectives of this article were to compile findings of relevant scientific papers, such as randomized controlled trials, systematic reviews, meta-analysis, guidelines and narrative reviews of literature, in order to promote knowledge about effectiveness of psychosocial interventions in eating disorders along time, in addition to showing the need for further research in specific areas.

## METHODS

### Type of study

#### Inclusion criteria

Randomized controlled trials (RCT), systematic reviews (SR), meta-analysis (MA), guidelines and reviews of literature on effectiveness of psychosocial interventions in eating disorders, including patients of any age and sex, with any chronic condition diagnosed together with eating disorders, according to Russell criteria (1979), the DSM and the International Classification of Diseases (ICD). Other study designs were included, such as prevention, cohort, cost-effectiveness, rapid response, pilot study, provided they were in accordance with the outcomes examined in this study. The data were extracted from abstract or full text, when necessary.

Other study designs, such as guidelines, cost-effectiveness ratio, prospective studies, risk factors, prevention, predictors and moderators of response to treatment were included whenever appropriate and relevant for the outcomes analyzed in this research (eating disorders symptoms, personal and social functioning, psychiatric co-morbidities, cognitive distortion, body image, adherence, weight).

The tables with the findings observed are divided into subtypes of eating disorders, and one classification of eating disorders in general. This division was done in order to facilitate the organization of research. In the general table, there are studies with the three types (AN, BN and BED) and subclinical forms.

#### Exclusion criteria

Papers mentioned above that examined other types of interventions (neither psychological nor psychosocial), such as diets, exercise, and medication.

## Types of intervention

### Experimental group

Interventions of all modalities and settings including psychological or psychosocial techniques, and their combinations among themselves or with medication.

### Control group

No treatment, waiting list, usual treatment (*e.g*. measuring weight and height, and nutritional counseling).

## Type of outcomes

### Primary outcome

Symptomatic remission: according to Russell (1979), DSM, ICD or standard scale (*e.g*. Eating Disorder Examination – EDE and Eating Disorders Examination Questionnaire - EDE-Q).

For AN: recovery of weight within the normal range (body mass index − BMI) at the end of therapy; for BN: 100% withdrawal from binge eating at the end of therapy, mean score of bulimic symptoms or frequency of binge eating at the end of treatment, weight BMI. For BED/eating disorder not otherwise specified (EDNOS), remission of bulimic symptoms, weight BMI.

Body image, cognitive distortion, psychological symptoms (anxiety, depression, obsessive compulsive symptoms), psychosocial functioning, and patient satisfaction.

Eating disorder symptom measurements using any recognized validated eating disorder questionnaire or interview schedule, *e.g*. the Morgan Russell Assessment Schedule (Morgan, 1988), Eating Attitudes Test (EAT; Garner, 1979), Eating Disorders Inventory (Garner, 1983; Garner, 1991).

### Secondary outcomes: adverse effects

**Search strategy**

An initial search was made in the Cochrane Database of Systematic Reviews (CDSR) of the Cochrane Library. The keywords used were “anorexia nervosa”, “bulimia nervosa”, “binge eating disorder”, “night eating syndrome” and “eating disorders”. All SR that included psychological or psychosocial interventions were included.

Later, the same keywords were searched at PubMed with limits of date (2001 to October 2013), considering that 2001 is the least recent update of CDSR, and type of studies: RCT, SR and MA. All primary studies, SR, MA and literature reviews addressing psychological or psychosocial interventions were included. Then, the literature after 2001 was searched based on types of eating disorders and outcomes, to provide an overview of evidence along time (before and after 2001), taking into account that the CDSR conducts an extensive search of primary studies, including unpublished literature, ongoing clinical trials and conference proceedings. Likewise, the PubMed database covers a considerable amount of scientific publications. Both searches allowed to have an overview of psychosocial techniques for eating disorders, based on studies published over time, as demonstrated throughout this article and especially in the tables of findings (Appendix 1).

We searched for additional data. In sources of guidelines, we searched: National Institute for Health and Clinical Excellence (NICE), Scottish Intercollegiate Guidelines Network (SIGN), Royal College of Physicians, Royal College of General Practitioners, Royal College of Nursing, NHS Evidence, Health Protection Agency, World Health Organization, National Guidelines Clearinghouse, Guidelines International Network, TRIP database, GAIN, NHS Scotland National Patient Pathways, New Zealand Guidelines Group, Agency for Healthcare Research and Quality (AHRQ), Institute for Clinical Systems Improvement (ICSI), National Health and Medical Research Council (Australia), Royal Australian College of General Practitioners (RACGP), British Columbia Medical Association, Canadian Medical Association (CMA), Alberta Medical Association, University of Michigan Medical School, Michigan Quality Improvement Consortium, Ministry of Health of Singapore, National Resource for Infection Control, Patient UK Guideline links, UK Ambulance Service Clinical Practice Guidelines, RefHELP NHS Lothian Referral Guidelines, MEDLINE (with guideline filter), Driver and Vehicle Licensing Agency and NHS Health at Work (occupational health practice).

As sources of health technology assessment and economic appraisals, we had: NIHR Health Technology Assessment programme, The Cochrane Library, NHS Economic Evaluations, Health Technology Assessments, Canadian Agency for Drugs and Technologies in Health and International Network of Agencies for Health Technology Assessment. As sources of RCT, we used The Cochrane Library, Central Register of Controlled Trials and MEDLINE (with RCT filter). Bandolier, Drug & Therapeutics Bulletin, TRIP database and Central Services Agency COMPASS Therapeutic Notes were sources of evidence-based reviews and evidence summaries; Department of Health and Health Management Information Consortium (HMIC) were sources of national policy.

### Review selection

The two authors independently assessed the titles and abstracts found in the Cochrane Database of Systematic Reviews of The Cochrane Library and at PubMed. Differences were resolved by discussion to reach consensus.

### Data analysis

The authors used the data extraction tables they prepared and analyzed each diagnosis of eating disorders separately, whenever possible, since the analyzed outcomes were different for each specific diagnosis. Data on population, interventions and outcomes were independently extracted and qualitatively analyzed. Differences were resolved by discussion to reach consensus. Individual narrative review summaries were used to present the results. A brief summary of the main findings was included in the discussion section. For more information on psychosocial techniques that are shown in the boxes, see the table of findings (Appendix 1).

## RESULTS

### The Cochrane Library

We identified five Cochrane SR on the treatment of eating disorders.^(6-10)^ The data of the last update and studies included in Cochrane SR are showed in table 1. The updates of the first version and subsequent versions of each Cochrane SR are done from time to time and may change or not the results of the current version. The main characteristics of the Cochrane SR are shown in appendix 2. The psychosocial interventions evaluated in Cochrane SR were self-help and guided self-help for eating disorders, family therapy and individual psychotherapy for AN, antidepressants *versus* psychological treatments (and their combination) for BN, and psychological treatments for BN and binging (binge eating). The results of the main outcomes analyzed are in appendix 3. The results of these five Cochrane SR are limited by their updates.

**Table 1:** Cochrane systematic reviews for treatment of eating disorders

Systematic review	Update	Number of studies	Number of participants	Studies
Anorexia nervosa				

Family therapy for those diagnosed with anorexia nervosa. Fisher et al.^(6)^	July 31, 2008	13	638	Hall, 1987; Russell, 1987; Crisp, 1991; le Grange, 1992; Robin, 1999; Eisler, 2000; Espina, 2000; Geist, 2000; Dare, 2001; Whitney unpublished, 2001; Ball, 2004; Lock, 2005; Rausch, 2006
Individual psychotherapy in the outpatient treatment of adults with anorexia nervosa. Hay et al.^(7)^	Feb 11, 2008	7	261	Channon, 1989; Treasure, 1995; Serfaty, 1999; Bachar, 1999; Dare, 2001; Bergh, 2002; McIntosh, 2005

Bulimia nervosa				

Antidepressants *versus* psychological treatments and their combination for bulimia nervosa. Hay et al.^(8)^	Aug 12, 2001	7	343	Mitchell, 1990; Fichter, 1991; Agras, 1992; Leitenberg, 1994; Russell, 1995b; Goldbloom, 1996; Walsh, 1997

Bulimia nervosa/binging				

Psychological treatments for bulimia nervosa and binging. Hay et al.^(9)^	May 31, 2007	48	3,054	Kirkley, 1985; Ordman, 1985; Wilson, 1986; Fairburn, 1986; Lee, 1986; Laessle, 1987; Freeman, 1988; Leitenberg, 1988; Bossert, 1989; Agras, 1989; Telch, 1990; Laessle, 1991; Fairburn, 1991; Wilson, 1991; Wolf, 1992; Griffiths, 1993; Garner, 1993; Wilfley, 1993; Thackwray, 1993; Agras, 1994; Cooper, 1995; Porzelius, 1995; Treasure, 1996; Walsh, 1997; Bulik, 1998; Peterson, 1998; Esplen, 1998; Carter, 1998; Bachar, 1999; Agras, 2000; Loeb, 2000; Nauta, 2000; Safer, 2001; Hsu, 2001; Kenardy, 2001; Telch, 2001; Wilfley, 2002; Sundgot-Borgen, 2002; Palmer, 2002; Durand, 2003; Ghaderi, 2003; Gorin, 2003; Carter, 2003; Bailer, 2003; Banasiak, 2005; Burton, 2006; Ljotsson, 2007; Munsch, 2007

Eating disorders				

Self-help and guided self-help for eating disorders. Perkins et al.^(10)^	May 23, 2006	15	1,191	Huon, 1985; Treasure, 1996; Thiels, 1998; Carter, 1998; Loeb, 2000; Mitchell, 2001; Palmer, 2002; Carter, 2003; Ghaderi, 2003; Durand, 2003; Walsh, 2004; Bailer, 2004; Grilo, 2005a; Grilo, 2005b; Banasiak, 2005

Total		90	5,487	

### Cochrane systematic reviews on anorexia nervosa

Family therapy showed reduction of symptoms after intervention in two short studies (lasting less than 12 months) as compared to usual care. When compared to psychological interventions, as cognitive behavior therapy (CBT), cognitive analytic group, ego-oriented psychotherapy, individual supportive therapy, no differences were found in four trials, as shown in appendix 3. However, in one study, that compared family therapy with individual supportive therapy, the participants were separated by age and duration of disease; significant results in remission of symptoms were found in younger people with an age of onset less than 18 years and less than 3 years of duration of disease with 21 participants (Group 1). The results were also significant in Group 1 for weight gain, but these two results were not significant at 5-year follow-up. In other Cochrane SR of psychotherapies for AN (not family therapy), there was insufficient evidence in comparisons to seven studies: focal psychoanalytic therapy, interpersonal psychotherapy (IPT), cognitive analytical therapy, cognitive therapy, CBT and behavioural therapy.

### Cochrane systematic reviews on bulimia nervosa and on binge eating disorder

#### Bulimia nervosa

When psychotherapy alone was compared to medication, there was better remission in five studies; the dropouts rates were higher in the antidepressant group in four studies as shown in appendix 3. The combined treatments compared to medication alone showed a better remission in the short run in four studies; also in combined treatments, the dropouts were higher for medication alone than combined treatments, in four studies. The combined treatments compared to psychotherapy alone showed a better remission; however, in combined treatments, the dropouts were higher than psychotherapy alone in six studies.

#### Bulimia nervosa and binge eating

The CBT (mainly CBT-BN) showed significant results in remission as compared to the waiting list/no treatment in 8 studies, as well as in mean bulimic symptoms in 12 studies. Besides, CBT showed improvement in depression symptoms as compared to the waiting list in seven studies, as shown in appendix 3. Comparing to others psychotherapies, IPT, behavioral therapy (BT), exposure and response prevention, hypnobehavioral therapy, supportive therapy, behavioral weight loss treatment, CBT was favored for remission symptoms in ten studies. When only studies of BN were considered, the difference was significant in seven studies. Moreover, when comparing CBT to others psychotherapies (all cited above plus non-directive counseling, supportive-expressive therapy, and weight loss therapy), CBT showed significant improvement in mean bulimic symptoms in 15 studies. Other psychotherapies showed some benefits, mainly IPT, in reducing binge eating in the long run as compared to no treatment. Self-help based in CBT approaches also resulted in some benefits. However, for weight variations, individual psychotherapy showed little or no reduction. Only BT for weight loss showed a trend in this direction in the subgroup of overweight patients and with BED.

## Cochrane systematic reviews on eating disorder

In Cochrane SR about pure self-help and guided self-help, the two types showed improvement as compared to waiting list for two studies about other eating disorder symptoms (no binging or purging), and to psychiatric symptoms and interpersonal functioning, as shown in appendix 3.

## PubMed database

The additional research was done at PubMed using the following MESH terms: “anorexia nervosa”, “bulimia nervosa”, “binge-eating disorder”, “night eating syndrome” and “eating disorders”. MA, RCT, SR and data range 2001-10/2013 were used as limits. A total of 716 studies were found. To include in the table of findings (Appendix 1), we selected 101 studies and 30 SR, MA or literature review (5 Cochrane SR). The results of searches and the number of studies included in the table of findings are shown in tables 2 and 3.

**Table 2:** Results of searches

Type	The Cochrane Library	PubMed (Since 2001)	Total
Primary studies	0	101	101*
Systematic reviews, meta-analysis, guidelines or literature reviews	5	25	30

* Six of these 101 studies were also included in the Cochrane systematic reviews (Dare, 2001; Bergh, 2002; Lock, 2005; Mc Intosh, 2005; Rausch, 2006; Munsch, 2007).

**Table 3:** Table of findings

Primary studies			

Anorexia nervosa	Bulimia nervosa	Binge eating disorder	Eating disorders
19	21	26	35
Total		101	

**Systematic reviews, meta-analysis, guidelines or literature reviews**			

**Anorexia nervosa**	**Bulimia nervosa**	**Binge eating disorder**	**Eaating disorders**

5 (2 CDSR)	4 (2 CDSR)	3	18 (1 CDSR)
Total		30	

CDSR: Cochrane Database of Systematic Reviews.

These tables include at total 101 studies and 30 SR, MA, guidelines, or literature review (5 Cochrane SR).

The findings were extracted from study abstracts. There were 19 studies and 5 SR, MA, guidelines or literature review for AN (2 Cochrane SR on psychotherapies); 21 studies and 4 SR, MA, guidelines or literature review for BN (2 Cochrane SR about psychotherapies for BN and binge eating); 26 studies and 3 SR, MA, guidelines or literature review for BED; and 35 studies and 18 SR, MA, guidelines or literature review for eating disorders in general (1 Cochrane SR on self-help). The overall results of the studies included are shown in the table of findings (Appendix 1).

## What works for eating disorders

Charts 1, 2 and 3 show which psychosocial interventions were tested in the included studies. The charts also display the techniques tested in each subtype of eating disorders, and overall, and the level of evidence presented at the time. Three boxes with subdivisions were made: (1) what works - interventions with consistent effectiveness; (2) what may work - interventions with some effectiveness; and (3) insufficient evidence - interventions that need more quality studies to test (to confirm or not) their effectiveness.

**Chart 1:** What works on psychosocial interventions for eating disorders

Anorexia nervosa
Maudsley model of family therapy for adolescents
Bulimia nervosa
CBT-BN for adults
Interpersonal psychotherapy
CBT-based self-help
CBT + fluoxetine
Eating disorders
CBT-based self-help
Enhanced cognitive behavior therapy/transdi agnostic CBT (CBT-E)
Binge eating disorder
CBT-BED for adults
CBT-based self-help
Interpersonal psychotherapy
Dialectical behavior therapy DBT-BED

CBT-BN: cognitive behavioral therapy for bulimia nervosa; CBT: cognitive behavioral therapy; CBT-E: enhanced cognitive behavior therapy; DBT-BED: dialectical behavior therapy for binge eating disorder.

**Chart 2:** What may work on psychosocial interventions for eating disorders

Anorexia nervosa
Family therapy for adolescents
Supportive psychoterapy
CBT for relapse prevention in adults
CBT-E for hospitalized adults
Focal psychoanalysis for adults
CBT + fluoxetine
Bulimia nervosa
Internet-based CBT
Manual-based CBT via telemedicine
Stepped care + CBT
Emotional and social mind training group
Eating disorders
Internet-delivered program for weight loss and eating disorders attitudes/behaviors in adolescents
Readiness and motivation therapy
CBT for weight management and eating disorders in children and adolescents
Interventions for treatment and prevention of body image and eating problems
Cognitive dissonance-based interventions
Psychoeducational training program in affection regulation
Dialectical behaviour therapy for concurrent eating disorders and substance abuse disorders
Short stepwise CBT for low self-esteem
Media literacy programs
Identity intervention programme to build new positive self-schemes
Children’s picture book to promote positive body image in young children
Mindfulness-based interventions
Binge eating disorder
Self-help based CBT
CBT delivery for overweight individuals with BED
Brief motivational interventions + self-help
Behavioural weight loss treatment
Combined treatments (CBT + medications – fluoxetine, topiramate, sertraline, orlistat)

CBT: Cognitive behavioral therapy; CBT-E: enhanced cognitive behavior therapy; BED: binge eating disorder.

**Chart 3:** Insufficient evidence of psychosocial interventions in eating disorders

Anorexia nervosa
Maudsley Model of Family Therapy for adults
Exposure therapy intervention focused on meal consumption
Bulimia nervosa
Appetite-focused dialectical behavior therapy
Eating disorder
Multidisciplinary care for all eating disorders in primary care
Longitudinal effects of media exposure of eating disorders symptoms
Binge eating disorder
Abstinence from binge eating and permanent weight loss

## DISCUSSION

One limitation of this article was the absence of a detailed methodological analysis of the investigations mentioned, due to the large amount of studies. Furthermore, it was necessary to summarize most results obtained. The complete material is displayed in the appendices.

On the other hand, this overview of scientific evidence on eating disorders provides a global approach of evidence along time (before and after 2001), taking into account that the CDSR include an extensive search of primary studies, including unpublished literature, ongoing clinical trials and conference proceedings. The same applies to PubMed, a database with high reliability and sensitivity. Moreover, since a mapping was done in two reliable databases - The Cochrane Library and PubMed, it is easy to perform an upgrade from the date of the search of studies on PubMed (10/2013) to get the update of new psychosocial techniques for eating disorders and learn about their progression over time.

### Anorexia nervosa

The effective psychotherapy in AN was short-duration family therapy, mainly the Maudsley family therapy for adolescents.^(11-21)^ In the Maudsley approach, the family plays a key role in recovery of patients with anorexia.^(12,13)^ For adolescents with severe obsessive-compulsive symptoms, the long-term family therapy can be more effective. When the parents clearly express much criticism towards the eating behavior of adolescents, it is recommended to avoid their presence in the initial sessions.^(19,20)^ For adults, the Maudsley family therapy should be better adapted in new studies.^(17)^ Some options of individual psychotherapies that may work for AN are focal psychoanalysis, CBT and supportive psychotherapy.^(18,21-24)^ In one multicenter study, the focal psychodynamics and CBT were compared in outpatients’ setting.^(25)^ Besides, the CBT may work for adults after hospital discharge to prevent relapse, and for inpatients with severe AN.^(22-26)^ The combination of CBT plus fluoxetine may help patients that already achieved a normal weight to maintain it.^(27)^ The best predictors found in a study of weight maintenance in weight-restored AN patients were the level of weight restoration after concluding the acute treatment and avoiding weight loss immediately after intensive treatment.^(27,28)^ In acute anorexia, low-dose antipsychotic medication may help, mainly for anxious and obsessive symptoms.^(29)^ An exposure therapy specifically focused on meal consumption was tested, but further studies are required to confirm its effectiveness.^(30)^

### Bulimia nervosa, binge eating disorder, and night eating syndrome

For BN, BED and the subclinical forms of these disorders, CBT is the most effective psychotherapy in reduction of associated behaviors, such as binge eating and purging.^(9,31-33)^ Adaptations of CBT were made especially for BN and BED (CBT-BN and CBT-BED).^(9,31)^ These approaches were made for adults, but they may be applied to older adolescents.^(15)^

The IPT is also effective to alleviate symptoms, mainly in the long run.^(9,31,33)^ The family therapy approaches showed benefits in bulimic adolescent patients, although they seem to be more effective in those with less associated psychopathology.^(24)^ Moroever, fluoxetine is effective to ameliorate of BN symptoms in the short run.^(8,24,32)^ The CBT can be offered in self-help (guided or not), and also can be addressed in different formats, such as computer software, CD-ROMs, internet, e-mail, telemedicine, telephone, short message service (SMS).^(34-41)^ Some strategies have been developed to increase effectiveness of psychosocial interventions, such as feedback after interventions, contact via SMS and text messaging.^(38,39)^ Besides, the dialectic BT is also effective for both (DBT-BN and DBT-BED).^(24,42,43)^ DBT is an approach that aims to reduce binge eating while improving adaptive emotion-regulation skills.^(24,42,43)^

The new “enhanced” version of the treatment (CBT-E) is an approach developed for all eating disorders and subclinical forms, drawn from the CBT-BN and taking into account transdiagnostic perspective of these disorders.^(2)^ This means that all eating disorders sharing the same core cognitive psychopathology − excessive value given to physical appearance and weight, which distinguishes them from other psychiatric disorders and is responsible for maintening eating disorders.^(2)^ It is called “enhanced” because it broadly describes strategies to increase compliance and have better treatment outcomes, dealing with some issues, such as humor intolerance, perfectionism, low self-esteem, and interpersonal difficulties.^(2)^

There is a growing number of studies addressing the association between weight control and eating disorders, especially in BED.^(34)^ Only the behavioral weight loss treatment may work for weight loss.^(9)^ Patients with BED do not generally have regular compensatory behaviors to combat excessive consumption of food, as BN patients do, and are often overweighted or obese.^(1)^ Due to the prevalence among eating disorders and strong association with obesity, BED is in DSM-V as a separate diagnostic category, and is no longer included in the EDNOS section, which facilitate its identification and treatment.^(1)^ The combination of psychosocial interventions and medications may be necessary to achieve both weight loss and reduced binge eating, and possible relief of depressive and anxiety symptoms. Some combined treatments include the following drugs: fluoxetine, topiramate, sertraline and orlistat.^(29,44)^ These combined treatments showed a reduction in weight loss in the short run, although there may be some side effects.^(29)^

Furthermore, the new constellation of eating symptoms that shows sufficient data in order to be included as a clinical condition in DSM-V, and that presents strong association with obesity is NES, as mentioned in the section of Feeding and Eating Conditions not Elsewhere Classified.^(1)^ NES is manifested by recurrent episodes of the night eating, like eating after awakening or excessive food consumption after the evening meal.^(1)^ Patients are aware of the episodes and recall them.^(1)^ NES is positively associated with stressful events, and the greater the degree of obesity, the greater the chance of having this syndrome.^(1)^ There is also indication of a significant correlation between NES and sleep disorders, anxiety and depression. For NES, a pilot study of CBT may work in decreasing the number of nocturnal ingestions and calorie intake after dinner.^(45)^ Behavioral strategies and brief relaxation also may work for reducing NES symptoms.^(45)^

### Eating disorders

Further studies about eating disorders are required, and they should address intervention and prevention techniques, in addition to risk factors, such as physical appearance, weight and eating concerns, as well as body image disturbance and internalization of media patterns, including both sexes and all age groups, since the current studies enrolled very few men and usually address older adolescents or adults.^(4,15)^

Furthermore, some studies were found on the construction of positive schemes on self-image and general aspects in female adults (and children and adolescents), that could be included in the school syllabus to prevent the development of eating disorders and mental dysfunctions related to body, eating behaviors and self-efficacy.^(4,15)^

Finally, it is necessary to dissemination the effective interventions for eating disorders offered by healthcare professionals, who are not specialized, aiming to promote multidisciplinary care, especially in primary health care. Many patients with eating disorders do not receive appropriate treatment or seek intervention for weight loss, in case of BN, BED and subclinical forms.^(4,9)^

## CONCLUSION

The studies included described the cognitive behavior approach as the most effective modality of psychological intervention. Others interventions that showed effectiveness were dialectical behavioral therapy, interpersonal therapy, family-based interventions and supportive therapies. The manual-based self-help is an intervention often effective and can be provide in different ways for prevention and treatment of eating disorders.

The binge eating disorder should be treated as a separate category of eating disorder, according to the DSM-V, and the night eating syndrome as a group of significant eating symptoms.

The effectiveness of psychosocial interventions for eating disorders may vary depending on the clinical features of patients, such as the level of chronicity and the biological and psychosocial co-morbidities. There is an increasing number of interventions that include eating disorder symptoms related to body image, concern about appearance and weight, self-esteem, as well anxiety and depression symptoms, which enhance applicability of these results in the clinical practice.

Taking into account the multifactorial etiology of eating disorders and the high prevalence of subclinical forms, the investigations are increasingly addressing interventions to prevent the development of these disorders by considering the individual, family and social risk factors. Yet, approaches that aim to build positive self-concept and self-image must be fostered.

For future research, it is important to report on knowledge about cognitive behavioral intervention techniques and other psychosocial approaches of eating disorders for different professionals, in various settings, in order to foster a multidisciplinary approach. Further studies analyzing cost-effectiveness of cognitive behavioral therapy and behavioral weight loss therapy are necessary. Investigations on psychosocial interventions for night eating syndrome are requires, since there are significant clinical eating symptoms. And, the impact of the media should be investigated in future longitudinal studies.
