# Supplementary material for: Effectiveness of psychosocial interventions in eating disorders: an overview of Cochrane systematic reviews
Source: Einstein (Sao Paulo). 2016 Apr-Jun;14(2):235–77. doi: 10.1590/S1679-45082016RW3120 (PMC4943360; doi:10.1590/S1679-45082016RW3120)
Supplement: Supplementary file 1 [file 1679-4508-eins-14-2-0235_sd.pdf]

Appendix 1. Table of findings - Anorexia nervosa

Study	Objective/methods	Main results	Conclusions
Dalle Grave R, Calugi S, Conti M, Doll H, Fairburn CG. Inpatient cognitive behaviour therapy for anorexia nervosa: a randomized controlled trial. Psychother Psychosom. 2013;82(6):390-8.	To compare the immediate and longer-term effects of two CBT programmes for hospitalized patients, one focused exclusively on the patients' ED features and the other focused also on mood intolerance, clinical perfectionism, core low self-esteem or interpersonal difficulties. Both programmes derived from CBT-E for ED. Eighty consecutive patients with severe AN were randomized to the two inpatient CBT-E programmes, each covering 20-week treatment.	Of the eligible patients, 81% accepted inpatient CBT-E, of whom 90% completed the 20-week treatment. The patients in both programmes showed significant improvements in weight, ED and general psychopathology. Deterioration after discharge did occur but it was not marked and was restricted to the first 6 months. There were no statistically significant differences between the effects of the two programme.	These findings suggest that both versions of inpatient CBT-E are well accepted by these severely-ill patients and might be a viable and promising treatment for severe anorexia nervosa. There appears to be no benefit from using the more complex form of the treatment.
Schmidt U, Oldershaw A, Jichi F, Sternheim L, Startup H, McIntosh V, et al. Out-patient psychological therapies for adults with anorexia nervosa: randomised controlled trial. Br J Psychiatry. 2012;201(5):392-9.	To evaluate the efficacy and acceptability of a novel psychological therapy for AN (MANTRA), and compared to the (SSCM). A total of 72 adult outpatients with AN or ED not otherwise specified were recruited from a specialist ED service in the UK.	At baseline, patients randomised to MANTRA were significantly less likely to be in a partner relationship than those receiving SSCM (3/34 *versus* 10/36; p<0.05). Patients in both treatments improved significantly in terms of ED and other outcomes, with no differences between groups. Strictly defined recovery rates were low. MANTRA patients were significantly more likely to require additional inpatient or daycare treatment than those receiving SSCM (7/34 *versus* 0/37; p=0.004).	Adults with AN are a difficult-to-treat group. The imbalance between groups in partner relationships may explain differences in service utilisation favouring SSCM. This study confirms SSCM as a useful treatment for outpatients with AN. The novel treatment, MANTRA, designed for this patient group may need adaptations to fully exploit its potential.
Godart N, Berthoz S, Curt F, Perdereau F, Rein Z, Wallier J, et al. A Randomized controlled trial of adjunctive family therapy and treatment as usual following inpatient treatment for anorexia nervosa adolescents. PLoS One. 2012;7(1):e28249.	To compare two multidimensional posthospitalization outpatients treatment programs for adolescents with severe AN: TAU *versus* this treatment plus TAU+FT.	At 18-month follow-up, we found a significant group effect for the Morgan and Russell outcome category in favor of the program with FT (intention-to-treat: TAU+FT: 12/30 (40%); TAU: 5/29 (17.2%); p=0.05; per protocol analysis: respectively 12/26 (46.2%); 4/27 (14.8%); p=0.01). Similar group effects were observed in terms of achievement of a healthy weight (*i.e.*, BMI≥ 10th percentile) and menstrual status.	Adding FT sessions, focusing on intrafamilial dynamics rather than eating symptomatology, to a multidimensional program improves treatment effectiveness in girls with severe AN.
Yu J, Stewart Agras W, Halmi KA, Crow S, Mitchell J, Bryson SW. A 1-year follow-up of a multi-center treatment trial of adults with anorexia nervosa. Eat Weight Disord. 2011;16(3):e177-81.	To examine maintenance of recovery following treatment in an adult AN population. One year follow-up of a randomized clinical trial with 122 participants treated with: CBT, drug therapy (fluoxetine), or a combination (CBT+fluoxetine) for 12 months. Participants were assessed at baseline, end of treatment, and follow-up.	A total of 52 participants completed the follow-up. Mean weight increased from end of treatment to follow-up. Of those who recovered weight at the end of treatment, 75% maintained this recovery at follow-up. Recovery of ED psychopathology was stable from end of treatment to follow-up, with 40% of participants with a global EDE score within normal range. Using the most stringent criteria for recovery, only 21% of the completer sample was recovered.	The findings suggest that while adults with AN improve with treatment and maintain these improvements during follow-up, the majority does not recover. Further research is needed to understand barriers to treatment completion and assessment.
Carter FA, Jordan J, McIntosh VV, Luty SE, McKenzie JM, Frampton CM, et al. The long-term efficacy of three psychotherapies for anorexia nervosa: a randomized, controlled trial. Int J Eat Disord. 2010;44(7):647-54.	To evaluate the long-term efficacy of three psychotherapies for AN. A total of 56 women with broadly defined AN who had participated in a RCT comparing specialized psychotherapies (CBT and IPT) to a control condition (SSCM), and attended long-term follow-up assessment (mean 6.7 years ±1.2).	Out of the original sample, 43% participated in long-term follow-up assessment (77%). Significantly different patterns of recovery were identified for the psychotherapies along time on the primary global outcome measure. Although SSCM was associated with a more rapid response than IPT, as per follow-up all three treatments were indistinguishable.	Potential implications for the timing of interventions to improve treatment response in AN are critically examined in this study.

continue...

...Continuation

Appendix 1. Table of findings - Anorexia nervosa

Study	Objective/methods	Main results	Conclusions
Lock J, Le Grange D, Agras WS, Moye A, Bryson SW, Jo B. Randomized clinical trial comparing family-based treatment with adolescent-focused individual therapy for adolescents with anorexia nervosa. Arch Gen Psychiatry. 2010;67(10):1025-32.	To evaluate the relative efficacy of FBT and AFT for adolescents with AN in full remission. A total of 121 participants, aged 12 to 18 years, with DSM-IV diagnosis of AN excluding the amenorrhea requirement. Twenty-four outpatient hours of treatment over 12 months of FBT or AFT. Participants were assessed at baseline, end of treatment, and at 6-month and 12-month follow-up after treatment.	There were no differences in full remission between treatments at end of treatment. However, at both the 6- and 12-month follow-up, FBT was significantly superior to AFT on this measure. FBT was significantly superior for partial remission at end of treatment, but not at follow-up. In addition, BMI percentile at end of treatment was significantly superior for FBT, but this effect was not found at follow-up. Participants in FBT also had greater changes in EDE score at end of treatment than those in AFT, but there were no differences at follow-up.	Although FBT and AFT led to considerable improvement and were similarly effective in producing full remission at end of treatment, FBT was more effective in facilitating full remission at both follow-up points.
Gowers SG, Clark AF, Roberts C, Byford S, Barrett B, Griffiths A, et al. A randomised controlled multicentre trial of treatments for adolescent anorexia nervosa including assessment of cost-effectiveness and patient acceptability - the TOuCAN trial. Health Technol Assess. 2010;14(15):1-98.	To evaluate the clinical effectiveness and cost-effectiveness of inpatient compared to outpatient treatment and general (routine) treatment in CAMHS against specialist treatment for young people with AN. A total of 167 young people (mean age 14 years 11 months) were randomised and 48 were followed up as a preference group. Randomised patients were allocated to either inpatient treatment in one of the four units with considerable experience in the treatment of AN, a specialist outpatient programme delivered in one of two centres, or TAU in general community CAMHS.	Of the 167 patients randomised, 65% adhered to the allocated treatment. Adherence was lower for inpatient treatment (49%) than for general CAMHS (71%) or specialist outpatient treatment (77%) (p=0.013). There was significant improvement in all groups at each time point, with the number achieving a good outcome being 19% at 1 year, 33% at 2 years and 64% (of those followed up) at 5 years. Analysis demonstrated no difference in treatment effectiveness of randomisation to inpatient compared with outpatient treatment, or specialist over generalist treatment at any time point, when baseline characteristics were taken into account. Specialist treatments had a higher probability of being more cost-effective than generalist treatments, and outpatient treatment had a higher probability of being more cost-effective than inpatient care. Parental satisfaction with treatment was generally good, though better with specialist than generalist treatment. Young people satisfaction was much more mixed, but again better with specialist treatment, including inpatient care.	This study provides little support for lengthy inpatient psychiatric treatment on clinical or health economic grounds. Outpatient care, supported by brief (medical) inpatient management for correction of acute complications may be a preferable approach. Comprehensive general CAMHS might, however, be well placed to manage milder cases. Further research should focus on the specific components of outpatient psychological therapies. Although family-based treatments are well established, trials have not established their effectiveness compared with good-quality individual psychological therapies and the combination of individual and family approaches is untested.
Roots P, Rowlands L, Gowers SG. User satisfaction with services in a randomised controlled trial of adolescent anorexia nervosa. Eur Eat Disord Rev. 2009;17(5):331-7.	To assess satisfaction of young people and parents with CAMHS outpatient, specialist outpatient and inpatient treatment received in a large randomised controlled trial. Quantitative and qualitative analysis of questionnaire data from 215 young people and their parents followed by focus groups to further explore emerging themes.	High levels of satisfaction were reported, more amongst parents than young people and with specialist services. Both young people and carers strongly valued clinical relationships that involved being listened to and understood. They valued the expertise of specialist rather than generic CAMHS services. There were polarised views on the influence of other young people in inpatient units. Parents in particular valued support for themselves, both from professionals and other parents, and felt this support for them and sibling was lacking.	All comprehensive CAMH services are able to provide the good generic psychotherapeutic skills that parents and young people value so highly. However, generic CAMHS struggle to provide the demanded level of expertise and more specialised individual and family therapeutic interventions.
Kaplan AS, Walsh BT, Olmsted M, Attia E, Carter JC, Devlin MJ, et al. The slippery slope: prediction of successful weight maintenance in anorexia nervosa. Psychol Med. 2009;39(6):1037-45.	To identify variables that predicted successful weight maintenance among weight-restored AN patients. A total of 93 patients with AN treated at two sites (Toronto and New York) through inpatient or partial hospitalization achieved a minimally normal weight and were then randomly assigned to receive fluoxetine or placebo along with CBT for 1 year. Clinical, demographic and psychometric variables were assessed after weight restoration prior to randomization and putative predictors of successful weight maintenance at 6 and 12 months were examined.	Higher BMI and lower rate of weight loss were associated with greater likelihood of maintaining a normal BMI at 6 and 12 months. An additional predictor of weight maintenance was site; patients in Toronto were better than those in New York. This study found that the best predictors of weight maintenance in weight-restored AN patients over 6 and 12 months were the level of weight restoration at the conclusion of acute treatment and the avoidance of weight loss immediately following intensive treatment.	These results suggest that outcome might be improved by achieving a higher BMI during structured treatment programs and on preventing weight loss immediately following discharge from such programs.

continue...

...Continuation

Appendix 1. Table of findings - Anorexia nervosa

Study	Objective/methods	Main results	Conclusions
Wild B, Friederich HC, Gross G, Teufel M, Herzog W, Giel KE, et al. The ANTOP study: focal psychodynamic psychotherapy, cognitive-behavioural therapy, and treatment-as-usual in outpatients with anorexia nervosa--a randomized controlled trial. Trials. 2009;10:23.	The aim of this multicentre study is to evaluate the efficacy of two standardized outpatient treatments for patients with AN: FPT and CBT. Each therapeutic approach is compared to a "treatment-as-usual" control group. A total of 237 patients meeting eligibility criteria are randomly and evenly assigned to the three groups - two intervention groups (CBT and FPT) and one control group. Body weight, eating-disorder related symptoms, and variables of therapeutic alliance are measured during the course of treatment.	Study protocol: the study design overcomes the disadvantages of previous studies, since it provides a randomized controlled design, a large sample size, adequate inclusion criteria, an adequate treatment protocol, and a clear separation of the treatment conditions in order to avoid contamination. Nevertheless, the study has to deal with difficulties specific to the psychopathology of AN. The treatment protocol allows for dealing with the typically occurring medical complications without dropping patients from the protocol.	
Gowers SG, Clark A, Roberts C, Griffiths A, Edwards V, Bryan C, et al. Clinical effectiveness of treatments for anorexia nervosa in adolescents: randomised controlled trial. Br J Psychiatry. 2007;191:427-35.	To evaluate the effectiveness of three readily available NHS treatments for adolescents (aged 12-18 years) with AN. Multicentre randomised controlled trial of 167 young people comparing inpatient, specialist outpatient and general CAMHS treatment.	Each group made considerable progress at 1 year, with further improvement by 2 years. Full recovery rates were poor (33% at 2 years, 27% still with AN). Adherence to inpatient treatment was only 50%. Neither inpatient nor specialist outpatient therapy demonstrated advantages over general CAMHS treatment by intention to treat, although some CAMHS outpatients were subsequently admitted on clinical grounds. Inpatient treatment (randomised or after outpatient transfer) predicted poor outcomes.	First-line inpatient psychiatric treatment does not provide advantages over outpatient management. Outpatient treatment failures do very poorly on transfer to inpatient facilities.
Steinglass J, Sysko R, Schebendach J, Broft A, Strober M, Walsh BT. The application of exposure therapy and D-cycloserine to the treatment of anorexia nervosa: a preliminary trial. J Psychiatr Pract. 2007;13(4):238-45.	To examine the utility and safety of an exposure therapy intervention and DCS in a population of patients with AN. Eleven participants completed a series of 6 laboratory meals, including pre- and post-exposure test meals and 4 exposure sessions. Participants were randomly assigned to receive either DCS or placebo in double-blind fashion before each of the 4 exposure sessions. These results were compared to data from a previously studied group of patients who received TAU.	Total calorie intake increased significantly from the baseline meal session to the post-test meal session in patients who received the exposure therapy intervention. Calorie intake did not increase significantly in the comparison group.	These data suggest that an exposure therapy intervention specifically focused on meal consumption may be helpful in increasing intake of a test meal.
Eisler I, Simic M, Russell GF, Dare C. A randomised controlled treatment trial of two forms of family therapy in adolescent anorexia nervosa: a five-year follow-up. J Child Psychol Psychiatry. 2007;48(6):552-60.	To ascertain the long-term impact of two forms of outpatient family intervention previously evaluated in a RCT. A five-year follow-up was conducted on a cohort of 40 patients who had received either CFT or SFT. All patients were traced and 38 agreed to be reassessed.	Overall there was little to distinguish in both treatments at 5 years, with more than 75% of subjects having no eating disorder symptoms. There were no deaths in the cohort and only 8% of those who had achieved a healthy weight by the end of treatment reported any kind of relapse. The only difference between the treatments was in patients from families with raised levels of maternal criticism. This group of patients had done less well at the end of treatment if they had been offered conjoint family meetings. At follow-up this difference was still evident, as shown in the relative lack of weight gain since the end of outpatient treatment.	This study confirms the efficacy of family therapy for adolescent AN, showing that those who respond well to outpatient family intervention generally stay well. The study provides further support for avoiding the use of conjoint family meetings, at least early on in treatment, when raised levels of parental criticism are evident.
Rausch Herscovici C. [Luch session, weight gain and their interaction with the psychopathology of anorexia nervosa in adolescents]. Vertex. 2006;17(65):7-15. Spanish.	To evaluate the effectiveness of the FMI on weight gain. Upon admission 12 adolescents diagnosed with AN were randomly assigned to two groups of outpatient family-based therapy, treated during 6 months, and followed-up at 12 months.	Both treatments produced considerable and comparable improvement in biological parameters, in depression, and in eating and general psychological symptoms. Although the FMI did not appear to have a significant effect on weight gain, results suggest it might have a differential benefit for the intractable self-starving patient with greater psychopathology.	The finding that weight recovery was not associated to general psychological outcome in those patients with more psychiatric co-morbidity, suggests caution when prioritizing clinical goals.

continue...

...Continuation

Appendix 1. Table of findings - Anorexia nervosa

Study	Objective/methods	Main results	Conclusions
Halmi KA, Agras WS, Crow S, Mitchell J, Wilson GT, Bryson SW, et al. Predictors of treatment acceptance and completion in anorexia nervosa: implications for future study designs. Arch Gen Psychiatry. 2005;62(7):776-81.	To evaluate factors leading to nonacceptance and noncompletion of treatment for 2 specific therapies and their combination in the treatment of AN. Randomized prospective study. A total of 122 patients meeting DSM-IV criteria for AN. Treatment with CBT, fluoxetine hydrochloride, or their combination for 1 year.	Of the 122 randomized cases, 21 (17%) were withdrawn; the overall dropout rate was 46% (56/122) in the remaining patients. Treatment acceptance occurred in 89 (73%) of the 122 randomized cases. Of the 41 assigned to medication alone, acceptance occurred in 23 (56%). In the other two groups, acceptance rate was differentiated by high and low obsessive preoccupation scores (rates of 91% and 60%, respectively). The only predictor of treatment completion was high self-esteem, which was associated with a 51% rate of treatment acceptance.	Acceptance of treatment and relatively high dropout rates pose a major problem for research in the treatment of AN. Differing characteristics predict dropout rates and acceptance, which need to be carefully studied before comparative treatment trials are conducted.
McIntosh VV, Jordan J, Carter FA, Luty SE, McKenzie JM, Bulik CM, et al. Three psychotherapies for anorexia nervosa: a randomized, controlled trial. Am J Psychiatry. 2005;162(4):741-7.	To examine the efficacy of CBT and IPT for AN. Fifty-six women with AN diagnosed by using strict and lenient weight criteria were randomly assigned to three treatments. Two were specialized psychotherapies (CBT and IPT), and one was a control treatment - NSCM.	For the total study group (intent-to-treat group), there were significant differences among therapies in the primary global outcome measure. NSCM was superior to IPT, while CBT was intermediate, neither worse than NSCM nor better than IPT. For the women completing therapy, NSCM was superior to the two specialized therapies.	The finding that NSCM was superior to more specialized psychotherapies (CBT and IPT) was opposite to the primary hypothesis and challenged assumptions about the effective ingredients of successful treatments for AN.
Lock J, Agras WS, Bryson S, Kraemer HC. A comparison of short- and long-term family therapy for adolescent anorexia nervosa. Acad Child Adolesc Psychiatry. 2005;44(7):632-9.	Research suggests that family treatment for adolescents with AN may be effective. This study was designed to determine the optimal length of such FT. A total of 86 adolescents (12-18 years of age) diagnosed with AN were allocated at random to either a short-term (10 sessions over 6 months) or long-term treatment (20 sessions over 12 months) and evaluated at the end of one year using the EDE.	An intent-to-treat analysis found no significant differences between the short-term and long-term treatment groups. *Post-hoc* analyses suggest that subjects with severe eating-related obsessive-compulsive features or who come from non-intact families respond better to long-term treatment.	A short-term course of family therapy appears to be as effective as a long-term course for adolescents with short-duration AN. However, there is a suggestion that those with more severe eating-related obsessive-compulsive thinking and non-intact families benefit from longer treatment.
Pike KM, Walsh BT, Vitousek K, Wilson GT, Bauer J. Cognitive behavior therapy in the posthospitalization treatment of anorexia nervosa. Am J Psychiatry. 2003;160(11):2046-9.	To evaluate CBT as a posthospitalization treatment for AN in adults. After hospitalization, 33 patients with DSM-IV AN were randomly assigned to one year of outpatient CBT or nutritional counseling.	The group receiving nutritional counseling relapsed significantly earlier and at a higher rate than the group receiving CBT (53% *versus* 22%). The overall treatment failure rate (relapse and dropping out combined) was significantly lower for CBT (22%) than for nutritional counseling (73%). The criteria for "good outcome" were met by significantly more of the patients receiving CBT (44%) than nutritional counseling (7%).	CBT was significantly more effective than nutritional counseling in preventing relapse in posthospitalization care of adult patients with AN.
**Systematic review/meta-analysis**	**Objective/methods**	**Main results**	**Conclusions**
Dare C, Eisler I, Russell G, Treasure J, Dodge L. Psychological therapies for adults with anorexia nervosa: randomised controlled trial of out-patient treatments. Br J Psychiatry. 2001;178:216-21.	To assess effectiveness of specific psychotherapies in outpatient management of adult patients with AN. Eighty-four patients were randomised to 4 treatments: 3 specific psychotherapies – (a) 1 year of FPP; (b) 7 months of CAT; (c) FT for 1 year; (d) low contact, "routine" treatment for 1 year (control).	At 1 year, there was symptomatic improvement in the whole group of patients. This improvement was modest, several patients being significantly undernourished at follow-up. FPP and FT were significantly superior to the control treatment; CAT tended to show benefits.	Psychoanalytic and family therapy are of specific value in the outpatient treatment of adult patients with anorexia.
Hartmann A, Weber S, Herpertz S, Zeeck A; German Treatment Guideline Group for Anorexia Nervosa. Psychological treatment for anorexia nervosa: a meta-analysis of standardized mean change. Psychother Psychosom. 2011;80(4):216-26. Review.	A review of all clinical trials available for ED was conducted, statistically integrating standardized mean change scores. After an extensive literature search, studies were selected, and classified by 3 independent raters. Weight gain - the main outcome criterion - was transformed into standardized mean change scores. Effect sizes were checked for homogeneity.	A total of 57 studies containing 84 treatment arms and 2,273 patients could be integrated. Studies differed considerably in quality. The strongest bias identified was reporting selectively on completers or failures, *versus* intention-to-treat samples. No significant differences between effect sizes could be identified concerning treatment setting, technique or patient characteristics. If treatment time is taken into account, inpatient treatment produced a faster weight gain than outpatient treatment.	The study describes weight gains which can be reached in outpatient and inpatient settings. It yielded no salient results speaking for a certain therapy technique, setting or procedure. Treatment guidelines for psychological treatment of AN still have to rely on lower level evidence.

continue...

...Continuation

Appendix 1. Table of findings - Anorexia nervosa

Systematic review/meta-analysis	Objective/methods	Main results	Conclusions
Oldershaw A, Hambrook D, Stahl D, Tchanturia K, Treasure J, Schmidt U. The socio-emotional processing stream in Anorexia Nervosa. Neurosci Biobehav Rev. 2011;35(3):970-88. Review.	This systematic review retrieved experimental studies of social-cognitive or affective processing in AN and categorised them using Ochsner's "social-emotional processing stream". Ochsner's "processing stream", based on healthy data, comprises 5 constructs: (1) acquisition of and (2) recognition and response to social-affective stimuli, (3) low-level and (4) high-level mental state inference, and (5) context-sensitive emotion regulation.	Thirty-seven experimental studies about AN were identified, mapping on to 4 of the 5 constructs (except construct 3). A meta-analysis of nine affect recognition studies was conducted. AN patients demonstrated impairments in all of the 4 domains with preliminary reports that some difficulties are trait-like, and others ameliorate following recovery.	Socio-emotional data was integrated with previous reports of neural abnormalities to generate an AN specific model of socio-emotional processing. Additional research is required for further definition and to translate experimental findings into clinical practice.
Fisher CA, Hetrick SE, Rushford N. Family therapy for anorexia nervosa. Cochrane Database Syst Rev. 2010;(4):CD004780. Review.	To evaluate the efficacy of FT compared with standard treatment and other treatments. RCTS of interventions described as "FT" compared to any other intervention or other types of FT were eligible for inclusion. Patients of any age or sex with a primary clinical diagnosis of AN were included.	Thirteen trials were included, the majority investigating family-based therapy, or variants. Reporting of trial conduct was generally inadequate. The full extent of the risk of bias is unclear. There was some evidence (from two studies, 81 participants) to suggest that FT may be more effective than TAU on rates of remission, in the short term. There was no significant advantage over other psychological interventions for FT based on 4 studies.	There is some evidence to suggest that FT may be effective compared to TAU in the short term. However, this is based on few trials that included only a small number of participants, all of which had issues regarding potential bias. There is insufficient evidence to be able to determine whether family therapy offers any advantage over other types of psychological interventions, or whether one type of family therapy is more effective than another. The field would benefit from a large, well-conducted trial.
Bulik CM, Berkman ND, Brownley KA, Sedway JA, Lohr KN. Anorexia nervosa treatment: a systematic review of randomized controlled trials. Int J Eat Disord. 2007;40:310–320. Review.	The RTI International-University of North Carolina at Chapel Hill Evidence-based Practice Center systematically reviewed evidence on efficacy of treatment for AN, harms associated with treatments, factors associated with treatment efficacy, and differential outcome by sociodemographic characteristics.	A total of 32 studies were included that involved only medications, only behavioral interventions, and medication plus behavioral interventions for adults or adolescents. The literature on medication treatments and behavioral treatments for adults with AN is sparse and inconclusive. CBT may reduce relapse risk for adults with AN after weight restoration, although its efficacy in the underweight state remains unknown. Variants of FT are efficacious in adolescents, but not in adults.	Evidence for AN treatment is weak; evidence for treatment-related harms and factors associated with efficacy of treatment are weak; and evidence for differential outcome by sociodemographic factors is nonexistent. Attention to sample size and statistical power, standardization of outcome measures, retention of patients in clinical trials, and developmental differences in treatment appropriateness and outcome is required.
Hay PP, Bacaltchuk J, Byrnes RT, Claudino AM, Ekmejian AA, Yong PY. Individual psychotherain the outpatient treatment of adults with anorexia nervosa. Database Syst Rev. 2003;(4):CD003909. Review.	To evaluate the evidence from randomised controlled trials for the efficacy of outpatient psychotherapies used in the treatment of older adolescents and adults with AN. All randomised controlled trials of adult individual outpatient therapy for AN, as defined by DSM-IV or similar international criteria.	Only 7 small trials were identified from the search, and aggregation of data was not possible. No specific treatment was consistently superior to any other specific approach. Dietary advice as a control arm had a 100% non-completion rate in one trial. One trial found a nonspecific therapy was favoured over 2 specific psychotherapies.	No specific approach can be recommended from this review. It is unclear why TAU performed so poorly, or why dietary advice alone appeared so unacceptable, as the reasons for non-completion were not reported.

CBT: cognitive behavior therapy; CBT-E: enhanced cognitive behaviour therapy; ED: eating disorders; AN: anorexia nervosa; MANTRA: Maudsley Model of Anorexia Nervosa Treatment for Adults; SSCM: Specialist Supportive Clinical Management; TAU: treatment as usual; TAU: treatment as usual plus family therapy; BMI: body mass index; EDE: Eating Disorder Examination; RCT: randomized controlled trials; IPT: interpersonal psychotherapy; FBT: family-based treatment; AFT: adolescent-focused individual therapy; DSM-IV: 4th ed. of the Diagnostic and Statistical Manual of Mental Disorders; CAMHS: Child and Adolescent Mental Health Services; FPT: focal psychodynamic therapy; NHS: National Health Service; DCS: D-cycloserine; CFT: conjoint family therapy; SFT: separated family therapy; FMI: family meal intervention; NSCM: nonspecific supportive clinical management; FT: family therapy; FPP: Focal psychoanalytic psychotherapy; CAT: cognitive-analytic therapy.

Extracted of PubMed
Table of findings - Bulimia nervosa

Study	Objective/methods	Main results	Conclusions
Wagner G, Penelo E, Wanner C, Gwinner P, Trofaier ML, Imgart H, et al. Internet-delivered cognitive-behavioural therapy vs. conventional guided self-help for bulimia nervosa: long-term evaluation of a randomised controlled trial. Br J Psychiatry. 2013;202:135-41.	To evaluate in a randomised controlled trial the long-term effectiveness of INT-GSH compared with BIB-GSH in females with BN. A total of 155 participants were randomly assigned to INT-GSH or BIB-GSH for 7 months. Outcomes were assessed at baseline, month 4, month 7 and month 18.	The greatest improvement was reported after 4 months with a continued reduction in ED symptoms reported at month 7 and 18. After 18 months, 14.6% (n=7/48) of the participants in the INT-GSH group and 25% (n=7/28) in the BIB-GSH group were abstinent from binge eating and compensatory measures, and 43.8% (n=21/48) and 39.2% (n=11/28) respectively were in remission. No differences regarding outcome between the two groups were found.	INT-GSH for BN was not superior as compared to BIB-GSH, the gold standard of self-help. Improvements remain stable in the long term.
Lavender A, Startup H, Naumann U, Samarawickrema N, Dejong H, Kenyon M, et al. Emotional and social mind training: a randomised controlled trial of a new group-based treatment for bulimia nervosa. PLoS One. 2012;7(10):e46047.	To test an ESM training group, against a CBT group CBT. treatment. Seventy-four participants were randomised to either ESM or CBT group treatment programmes. All participants were offered 13 group and 4 individual sessions.	There were no differences in outcome between the two treatments. No moderators of treatment outcome were identified. Adherence rates were higher for participants in the ESM group.	This suggests that ESM may be a viable alternative to CBT for some individuals. Further research will be required to identify and preferentially allocate suitable individuals accordingly.
McIntosh VV, Carter FA, Bulik CM, Frampton CM, Joyce PR. Five-year outcome of cognitive behavioral therapy and exposure with response prevention for bulimia nervosa. Psychol Med. 2011;41(5):1061-71.	To examine the longer-term outcome of BN following treatment with CBT and ERP. A total of 135 women with purging BN received eight sessions of individual CBT and were then randomly assigned to either relaxation training or one of two ERP treatments, B-ERP or P-ERP cues P-ERP. Participants were assessed yearly following treatment and follow-up data were recorded.	Eighty-one per cent of the total sample attended long-term follow-up. At 5 years, abstinence rates from binging were significantly higher for the two exposure treatments (43 and 54%) than for relaxation (27%), with no difference between the two forms of exposure. Over 5 years, the frequency of purging was lower for the exposure treatments than for relaxation training. Rates of recovery varied according to definition of recovery. Recovery continued to increase to 5 years. At 5 years, 83% no longer met DSM-III-R criteria for BN, 65% received no eating disorder diagnosis, but only 36% had been abstinent from bulimic behaviors for the past year.	This study provides possible evidence of a conditioned inoculation from exposure treatment compared with relaxation training in long-term abstinence from binge eating at 5 years, and the frequency of purging over 5 years, but not for other features of BN. Differences among the groups were not found prior to 5 years. CBT is effective for BN, yet a substantial group remains unwell in the long term. Definition of recovery impacts markedly on recovery rates.
Mitchell JE, Agras S, Crow S, Halmi K, Fairburn CG, Bryson S, et al. Stepped care and cognitive-behavioural therapy for bulimia nervosa: randomised trial. Br J Psychiatry. 2011;198(5):391-7.	This study compared CBT augmented by fluoxetine if indicated, with a stepped-care treatment approach for BN. This was a RCT conducted at four clinical centres. Randomization of 293 participants with BN to one of two treatment conditions: manual-based CBT delivered in an individual therapy format involving 20 sessions over 18 weeks and participants who were predicted to be non-responders after 6 sessions of CBT had fluoxetine added to treatment; or a stepped-care approach that began with supervised self-help, with the addition of fluoxetine in participants who were predicted to be non-responders after six sessions, followed by CBT for those who failed to achieve abstinence with self-help and medication management.	Both in the intent-to-treat and completer samples, there were no differences between the two treatment conditions in inducing recovery (no binge eating or purging behaviours for 28 days) or remission (no longer meeting DSM-IV criteria). At the end of 1-year follow-up, the stepped-care condition was significantly superior to CBT.	Therapist-assisted self-help was an effective first-level treatment in the stepped-care sequence, and the full sequence was more effective than CBT suggesting that treatment is enhanced with a more individualised approach.
Hill DM, Craighead LW, Safer DL. Appetite-focused dialectical behavior therapy for the treatment of binge eating with purging: a preliminary trial. Int J Eat Disord. 2011;4(3):249-61.	This treatment development study investigated the acceptability and efficacy of a modified version of DBT for BN, entitled "appetite focused DBT" (DBT-AF). Thirty-two women with binge/purge episodes at least one time per week were randomly. assigned to 12 weekly sessions of DBT-AF (n=18) or to a 6-week delayed treatment control (n=14).	Treatment attrition was low, and DBT-AF was rated highly acceptable. At 6 weeks, participants who were receiving DBT-AF reported significantly fewer BN symptoms than controls. At post-test, 26.9% of the 26 individuals who entered treatment (18 initially assigned and 8 from the delayed treatment control) were abstinent from binge/purge episodes for the past month; 61.5% no longer met full or subthreshold criteria for BN. Participants demonstrated a rapid rate of response to treatment and achieved clinically significant change.	Results suggest that DBT-AF warrants further investigation as an alternative to DBT or CBT for BN.

continue...

...Continuation

Extracted of PubMed

Table of findings - Bulimia nervosa

Study	Objective/methods	Main results	Conclusions
Sánchez-Ortiz VC, Munro C, Stahl D, House J, Startup H, Treasure J, et al. A randomized controlled trial of internet-based cognitive-behavioural therapy for bulimia nervosa or related disorders in a student population. Psychol Med. 2011;41(2):407-17.	Bulimic ED are common among female students, yet the majority do not access effective treatment. iCBT may be able to bridge this gap. A total of 76 students with BN or EDNOS were randomly assigned to immediate iCBT with e-mail support over 3 months, or to a 3-month waiting list followed by iCBT (WL/DTC).	Students who had immediate iCBT showed significantly greater improvements at 3 and 6 months than those receiving WL/DTC in ED and other symptoms.	iCBT with e-mail support is efficacious in students with bulimic disorders and has lasting effects.
Katzman MA, Bara-Carril N, Rabe-Hesketh S, Schmidt U, Troop N, Treasure J. A randomized controlled two-stage trial in the treatment of bulimia nervosa, comparing CBT versus motivational enhancement in Phase 1 followed by group versus individual CBT in Phase 2. Psychosom Med. 2010;72(7):656-63.	To conduct a randomized, controlled, two-stage trial in the treatment of BN, comparing CBT compaired motivational enhancement in phase 1, followed by group versus individual CBT in phase 2. All together, 225 patients with BN or EDNOS were recruited into a RCT lasting 12 weeks with follow-ups at 1 year and 2.5 years.	Patients improved significantly across all of the interventions with no differences in outcome or treatment adherence. Including motivational enhancement therapy rather than a CBT first phase of treatment did not affect outcome.	Outcome differences between individual and group CBT were minor, suggesting that group treatment preceded by a short individual intervention may be a cost-effective alternative to purely individual treatment.
Johnston O, Startup H, Lavender A, Godfrey E, Schmidt U. Therapeutic writing as an intervention for symptoms of bulimia nervosa: effects and mechanism of change. Int J Eat Disord. 2010;43(5):405-19.	To explore the effects on bulimic symptomatology of a writing task intended to reduce emotional avoidance. Eighty individuals reporting symptoms of bulimia completed, by e-mail, a therapeutic or control writing task. Participants completed questionnaires on bulimic symptoms, mood, and potential moderating and mediating factors, and were followed up after 4 and 8 weeks. Writing content was explored using a word count package and qualitative framework analysis.	Bulimic symptoms decreased in both groups, although the number of participants who improved was approximately equal to the number who did not improve in the two groups. Symptom reduction was associated with increases in perceived mood regulation abilities, and decreases in negative beliefs about emotions. Participants preferred internet delivery to face-to-face discussion.	For individuals experiencing symptoms of bulimia, the effects of therapeutic writing did not differ significantly from effects of a control writing task.
Schützmann K, Schützmann M, Eckert J. [The efficacy of outpatient client-centered psychotherapy for bulimia nervosa: results of a randomised controlled trial]. Psychother Psychosom Med Psychol. 2010;60(2):52-63. German.	The efficacy of outpatient CCP for BN was investigated using GSH as comparison group. In the initial CCP-group (n=29), 27,6% still met the diagnostic criteria at end of treatment and 34,5% at follow-up.	In the completer sample n=25), 16% met the diagnostic criteria at end of treatment and 0% at follow-up. Significant improvements in eating behaviour, comorbid and general psychopathology could be demonstrated and persisted over the follow-up period; in part, they were significantly superior to the improvements in the GSH-group. As regards the diagnostic criteria, the CCP proved to be significantly more effective than the GSH for both the ITT and the completer samples. At follow-up, this group difference was present in the completer samples only.	The results provide initial evidence for the efficacy of CCP in the outpatient treatment of women with BN.
Crow SJ, Mitchell JE, Crosby RD, Swanson SA, Wonderlich S, Lancanster K. The cost effectiveness of cognitive behavioral therapy for bulimia nervosa delivered via telemedicine versus face-to-face. Behav Res Ther. 2009;47(6):451-3.	To examine the cost-effectiveness of telemedicine delivery of CBT for BN. A RCT of face-to-face *versus* telemedicine CBT for BN. Randomization of 128 women with DSM-IV BN or EDNOS subsyndromal variants of BN, to 20 sessions of treatment over 16 weeks. A cost-effectiveness analysis from a societal perspective was conducted.	The total cost per recovered (abstinent) subject was US$9324.68 for face-to-face CBT, and US$7300.40 for telemedicine CBT. The cost differential was accounted for largely by therapist travel costs. Sensitivity analyses examining the costs of therapy sessions, gasoline and telemedicine connection yielded fundamentally similar results.	In this study, CBT delivered face-to-face and via telemedicine were similarly effective, and telemedicine delivery cost was substantially less. These findings underscore the potential applicability of telemedicine approaches to ED treatment and psychiatric treatment in general.
Steele AL, Wade TD. A randomised trial investigating guided self-help to reduce perfectionism and its impact on bulimia nervosa: a pilot study. Behav Res Ther. 2008;46(12):1316-23.	To evaluate reducing perfectionism as a potential treatment target for individuals with BN. Forty-eight individuals meeting DSM-IV criteria for BN or EDNOS with binge eating (objective or subjective) or purging at least once per week were recruited. Participants were randomly assigned to receive 8 sessions of manual-based GSH over a 6-week period that was focused on either CBT for perfectionism, CBT for BN, or a placebo. Individuals were assessed at baseline, pre-treatment, post-treatment and at 6-month follow-up on 12 outcome variables, including diagnostic criteria and psychological variables.	There was no significant change in any of the outcome variables over a 6-week no-treatment period, but at post-treatment and 6-month follow-up there were significant main effects of time for ten and eight outcome variables respectively. This suggests that all groups reported significant reductions in bulimic symptomatology and related psychopathology at post-treatment and follow-up.	These findings show potential for the use of novel interventions in GSH for BN.

continue...

...Continuation

Extracted of PubMed

Table of findings - Bulimia nervosa

Study	Objective/methods	Main results	Conclusions
Schmidt U, Andiappan M, Grover M, Robinson S, Perkins S, Dugmore O, et al. Randomised controlled trial of CD-ROM-based cognitive-behavioural self-care for bulimia nervosa. Br J Psychiatry. 2008;193(6):493-500.	To examine the effectiveness of a CD-ROM-based cognitive-behavioural intervention in BN and EDNOS (bulimic type) in a routine setting. Ninety-seven people with BN or EDNOS were randomised to either CD-ROM without support for 3 months followed by a flexible number of therapist sessions or to a 3-month waiting list followed by 15 sessions of therapist CBT. Clinical symptoms were assessed at pre-treatment, 3 months and 7 months.	Only two-thirds of participants started treatment. Although there were significant group *versus* time interactions for bingeing and vomiting, favouring the CD-ROM group at 3 months and the waiting-list group at 7 months, *post-hoc* group comparisons at 3 and 7 months found no significant differences for bingeing or vomiting.	CD-ROM-based delivery of this intervention, without support from a clinician, may not be the best way of exploiting its benefits.
Mitchell JE, Crosby RD, Wonderlich SA, Crow S, Lancaster K, Simonich H, et al. A randomized trial comparing the efficacy of cognitive-behavioral therapy for bulimia nervosa delivered via telemedicine versus face-to-face. Behav Res Ther. 2008;46(5):581-92.	To compare the relative efficacy and acceptability of a manual-based CBT for BN delivered in person to a comparable therapy delivered via telemedicine. A total of 128 adults meeting DSM-IV criteria for BN or EDNOS with binge eating or purging at least once per week were recruited through referrals from clinicians and media advertisements in the targeted geographical areas. Participants were randomly assigned to receive 20 sessions of Manual-based, CBT for BN over 16 weeks delivered either face-to-face (FTF-CBT) or via telemedicine (TV-CBT) by trained therapists. The primary outcome measures were binge eating and purging frequency as assessed by interview at the end of treatment, and again at 3- and 12-month follow-ups.	Retention in treatment was comparable for TV-CBT and FTF-CBT. Abstinence rates at end-of-treatment were generally slightly higher for FTF-CBT as compared with TV-CBT, but the differences were not statistically significant. FTF-CBT patients also experienced significantly greater reductions in eating disordered cognitions and interview-assessed depression. However, the differences overall were few in number and of marginal clinical significance.	CBT for BN delivered via telemedicine was both acceptable to participants and roughly equivalent in outcome to therapy delivered in person.
Le Grange D, Crosby RD, Lock J. Predictors and moderators of outcome in family-based treatment for adolescent bulimia nervosa. J Am Acad Child Adolesc Psychiatry. 2008;47(4):464-70.	To explore the predictors and moderators of treatment outcome for adolescents with BN who participated in FBT or individual supportive psychotherapy Data derived from a RCT (n=80) of FBT of BN and supportive psychotherapy were used to explore possible predictors and moderators of treatment outcome.	Participants with less severe EDE eating concerns at baseline were more likely to have remitted (abstained from binge eating and purging) after treatment (odds ratio - OR of 0.47; $p<0.01$) and follow-up (OR of 0.53; $p<0.01$), regardless of the treatment that they received. Participants with lower baseline depression scores were more likely to have partial remission (no longer meeting study entry criteria) after treatment (OR of 0.93; $p<0.01$), whereas those with fewer binge-eating/purging episodes at baseline were more likely to have partial remission at follow-up (OR of 0.98; $p<0.05$). In terms of moderators, participants with less severe eating disorder psychopathology, receiving FBT-BN, were more likely to meet criteria for partial remission at follow-up (OR of 0.44; $p<0.05$).	Lower eating concerns are the best predictor of remission for adolescents with BN, and FBT of BN may be most effective in those cases with low levels of ED psychopathology.
le Grange D, Crosby RD, Rathouz PJ, Leventhal BL. A randomized controlled comparison of family-based treatment and supportive psychotherapy for adolescent bulimia nervosa. Arch Gen Psychiatry. 2007;64(9):1049-56.	To evaluate the relative efficacy of FBT and SPT for adolescents with BN. A total of 80 patients, aged 12 to 19 years, with a DSM-IV diagnosis of BN or a strict definition of partial BN. The interventions were 20 outpatient visits over 6 months of FBT or SPT. Participants were followed up at 6 months after treatment. The main outcome was abstinence from binge-and-purge episodes as measured by the EDE.	Forty-one patients were assigned to FBT and 39 to SPT. Categorical outcomes at post-treatment demonstrated that significantly more patients receiving FBT (16; 39%) were binge-and-purge abstinent compared with those receiving SPT (7; 18%; $p=0.049$). Fewer patients were abstinent at the 6-month follow-up; however, the difference was statistically in favor of FBT *versus* SPT (12 patients [29%] *versus* 4 patients [10%]; $p=0.05$). Secondary outcome assessment, based on random regression analysis, revealed main effects in favor of FBT on all measures of eating pathological features ($p=0.003$ to $p=0.03$ for all).	FBT showed a clinical and statistical advantage over SPT at post-treatment and at 6-month follow-up. Reduction in core bulimic symptoms was also more immediate for patients receiving FBT *versus* SPT.

continue...

...Continuation

Extracted of PubMed

Table of findings - Bulimia nervosa

Study	Objective/methods	Main results	Conclusions
Schmidt U, Lee S, Beecham J, Perkins S, Treasure J, Yi I, et al. A randomized controlled trial of family therapy and cognitive behavior therapy guided self-care for adolescents with bulimia nervosa and related disorders. Am J Psychiatry. 2007;164(4):591-8.	To compare the efficacy and cost-effectiveness of FT and CBT guided self-care in adolescents with BN or EDNOS. Eighty-five adolescents were randomly assigned to FT for BN or individual CBT guided self-care supported by a healthcare professional. The primary outcome measures were abstinence from binge-eating and vomiting, as assessed by interview at end of treatment (6 months) and again at 12 months.	Of the 85 study participants, 41 were assigned to FT and 44 to CBT guided self-care. At 6 months, bingeing had undergone a significantly greater reduction in the guided self-care CBT group than in the FT group; however, this difference disappeared at 12 months. There were no other differences between groups in behavioral or attitudinal eating disorder symptoms. The direct cost of treatment was lower for guided self-care than for FT. The two treatments did not differ in other cost categories.	Compared with FT, CBT guided self-care has the slight advantage of offering a more rapid reduction of bingeing, lower cost, and greater acceptability for adolescents with BN or EDNOS.
Burton E, Stice E. Evaluation of a healthy-weight treatment program for bulimia nervosa: a preliminary randomized trial. Behav Res Ther. 2006;44(12):1727-38.	To test whether healthy dieting maintains bulimic symptoms or effectively reduces this eating disturbance. Female participants (n=85) with full- and sub-threshold BN were randomly assigned to a 6-session healthy dieting intervention or waiting-list condition and assessed through 3-month follow-up.	Relative to control participants, intervention participants showed modest weight loss during treatment and demonstrated significant improvements in bulimic symptoms that persisted through follow-up.	These preliminary results suggest that this intervention shows potential for the treatment of BN and may be worthy of future refinement and evaluation. Results also provide experimental evidence that dieting behaviors do not maintain BN, suggesting the need to reconsider maintenance models for this eating disorder.
Schmidt U, Landau S, Pombo-Carril MG, Bara-Carril N, Reid Y, Murray K, et al. Does personalized feedback improve the outcome of cognitive-behavioural guided self-care in bulimia nervosa? A preliminary randomized controlled trial. Br J Clin Psychol. 2006;45(Pt 1):111-21.	To evaluate the addition of personalized feedback to a guided CBT self-help programme for patients with BN. Sixty-one patients with DSM-IV BN or EDNOS were randomly allocated to receive 14 sessions of CBT guided self-care with or without added personalised feedback on current physical and psychological status, risk and problems, and variables facilitating or hindering change. Feedback to patients was delivered in a number of ways: (a) personalised letters after assessment and at the end of treatment, (b) a specially designed feedback form administered half-way through treatment, (c) computerised feedback about bulimic and other symptoms, such as anxiety, depression and interpersonal functioning repeated at intervals throughout treatment and follow-up.	Outcome was assessed using patient-rated measures of bulimic symptoms at the end of treatment and at 6-month follow-up. The data were analysed using maximum likelihood methods to assess group differences at the follow-up. Added feedback did not have an effect on take-up or drop-out from treatment. However, it improved outcome by reducing self-induced vomiting and dietary restriction more effectively.	The findings lend support to the notion that the addition of repeated personalised feedback improves outcome from guided CBT self-help treatment and deserves further study.
Ghaderi A. Does individualization matter? A randomized trial of standardized (focused) versus individualized (broad) cognitive behavior therapy for bulimia nervosa. Behav Res Ther. 2006;44(2):273-88.	Does higher level of individualization increase treatment efficacy? Fifty patients with BN were randomized into either manual-based (focused) or more Individualized (broader) CBT guided by logical functional analysis. EDE and a series of self-report questionnaires were used for assessment at pre-, and post-treatment, as well as at follow-up.	Both conditions improved significantly at post-treatment, and the results were maintained at 6-month follow-up. There were no statistically and clinically significant differences between the two conditions at post-treatment, with the exception of abstinence from objective bulimic episodes, eating concerns, and body shape dissatisfaction, all favoring the individualized, broader condition. Both groups improved concerning self-esteem, perceived social support from friends, and depression. The improvements were maintained at follow-up. Ten patients (20%) did not respond to treatment. Notably, a majority of non-responders (80%) were in the manual-based condition. Non-responders showed extreme dominance of rule-governed behavior, and lack of contact with actual contingencies compared to responders.	The study provided preliminary support for the superiority of higher level of Individualization (*i.e.* broader CBT) in terms of the response to treatment, and relapses. However, the magnitude of effects was moderate, and independent replications, with blind assessment procedures, and a larger sample sized are needed before more clear-cut conclusions can be drawn.

continue...

...Continuation

Extracted of PubMed

Table of findings - Bulimia nervosa

Study	Objective/methods	Main results	Conclusions
Carrard I, Rouget P, Fernández-Aranda F, Volkart AC, Damoiseau M, Lam T. Evaluation and deployment of evidence-based patient self-management support program for Bulimia Nervosa. Int J Med Inform. 2006;75(1):101-9.	This article presents initial results from an European multicentre study to determine the effectiveness and feasibility of an online Self-help treatment support program for BN. The on-line program is based on CBT and consists of seven steps that patients work through progressively. A total of 141 women suffering from BN used the program over a 6-month period. Patients were supported by three face-to-face evaluation interviews with a therapist, and a weekly e-mail contact.	Initial results from the Swiss sample (n=41) showed significant improvement of overall psychological health (p<0.001) as measured by the SCL-90R, and for all dimensions of the EDI-2.	An online self-help program for BN can be used effectively to reduce eating disorder symptoms in bulimic patients, and user feedback showed that this approach contributed to increase patient involvement and service availability. Additional data from the other centers will further inform the efficacy and impact of this approach.
Bergh C, Brodin U, Lindberg G, Södersten P. Randomized controlled trial of a treatment for anorexia and bulimia nervosa. Proc Natl Acad Sci USA. 2002;99(14):9486-91.	A total of 16 patients, randomly selected out of a group composed of 19 patients with AN and 13 with BN, were trained to eat and recognize satiety by using computer support. They rested in a warm room after eating, and their physical activity was restricted. The patients in the control group (n=16) received no treatment. Remission was defined by normal body weight (anorexia), cessation of binge eating and purging (bulimia), a normal psychiatric profile, normal laboratory test values, normal eating behavior, and resumption of social activities.	Fourteen patients went into remission after a median of 14.4 months (range 4.9-26.5) of treatment, but only one patient went into remission while waiting for treatment (p=0.0057). Relapse is considered a major problem in patients who have been treated to remission. We therefore report results on a total of 168 patients who have entered our treatment program. The estimated rate of remission was 75%, and estimated time to remission was 14.7 months (quartile range 9.6 > or = 32). Six patients (7%) out of 83 who were treated to remission relapsed, but the others (93%) have remained in remission for 12 months (quartile range 6-36).	Because the risk of relapse is maximal in the first year after remission, we suggest that most patients treated with this method recover.
**Systematic review/meta-analysis**	**Objective/methods**	**Main results**	**Conclusions**
Zakzanis KK, Campbell Z, Polsinelli A. Quantitative evidence for distinct cognitive impairment in anorexia nervosa and bulimia nervosa. J Neuropsychol. 2010;4(Pt 1):89-106. Review.	To articulate the magnitude of Cognitive Impairment in patients with AN and BN by quantitatively synthesizing the existing literature using meta-analytic methodology.	The results demonstrate modest evidence of cognitive impairment specific to AN and BN that is related to body mass index in AN in terms of its severity, and is differentially impaired between disorders.	Together, these results suggest that disturbed cognition is figural in the presentation of ED and may serve to play an integral role in its cause and maintenance.
Hay PP, Bacaltchuk J, Stefano S, Kashyap P. Psychological treatments for bulimia nervosa and binging. Cochrane Database Syst Rev. 2009;(4):CD000562. Review.	To evaluate the efficacy of CBT, CBT-BN and other psychotherapies in the treatment of adults with BN or related syndromes of recurrent binge eating. Randomised controlled trials of psychotherapy for adults with BN, binge eating disorder and/or EDNOS of a bulimic type which applied a standardised outcome methodology and had less than 50% dropout rate.	A total of 48 studies (n=3,054 participants) were included. The review supported the efficacy of CBT and particularly CBT-BN in the treatment of people with BN and also (but less strongly due to the small number of trials) related ED syndromes. Other psychotherapies were also efficacious, particularly interpersonal psychotherapy in the longer-term. Self-help approaches that used highly structured CBT treatment manuals were promising. Exposure and response prevention did not enhance the efficacy of CBT. Psychotherapy alone is unlikely to reduce or change body weight in people with BN or similar eating disorders.	There is a small body of evidence for the efficacy of CBT in BN and similar syndromes, but the quality of trials varies a lot and sample sizes are often small. More and larger trials are needed, particularly for binge eating disorder and other EDNOS syndromes. There is a need to develop more efficacious therapies for those with both a weight and an ED.

continue...

...Continuation

Extracted of PubMed

Table of findings - Bulimia nervosa

Systematic review/meta-analysis	Objective/methods	Main results	Conclusions
Shapiro JR, Berkman ND, Brownley KA, Sedway JA, Lohr KN, Bulik CM. Bulimia nervosa treatment: a systematic review of randomized controlled trials. Int J Eat Disord. 2007;40(4):321-36. Review.	The RTI-UNC EPC systematically reviewed evidence on efficacy of treatment for BN, harms associated with treatments, factors associated with treatment efficacy, and differential outcome by sociodemographic characteristics.	Forty-seven studies with 4,787 participants of medication only, behavioral interventions only, and medication plus behavioral interventions for adults or adolescents met our inclusion criteria. Fluoxetine (60mg/day) decreases the core symptoms of binge eating and purging and associated psychological features in the short term. CBT reduces core behavioral and psychological features in the short- and long-term.	Evidence for medication or behavioral treatment for BN is strong, for self-help is weak; for harms related to medication is strong but either weak or nonexistent for other interventions; and evidence for differential outcome by sociodemographic factors is nonexistent. Attention to sample size, standardization of outcome measures, attrition, and reporting of abstinence from target behaviors are required. Longer follow-up intervals, innovative treatments, and attention to sociodemographic factors would enhance the literature.
Hay PP, Claudino AM, Kaio MH. Antidepressants versus psychological treatments and their combination for bulimia nervosa. Cochrane Database Syst Rev. 2001;(4):CD003385. Review.	To conduct a systematic review of all RCT comparing antidepressants with psychological approaches or comparing their combination with each single approach for the treatment of BN. Inclusion criteria: every RCT in which antidepressants were compared with psychological treatments or the combination of antidepressants with psychological approaches was compared to each treatment alone, to reduce the symptoms of BN in patients of any age or sex.	Five trials were included in comparison. One (antidepressants *versus* psychological treatments), five in Comparison Two (antidepressants *versus* the combination) and seven in Comparison Three (psychological treatments versus the combination). Remission rates were 20% for single antidepressants compared to 39% for single psychotherapy. Dropout rates were higher for antidepressants than for psychotherapy. Comparison Two found remission rates of 42% for the combination *versus* 23% for antidepressants. Comparison Three showed a 36% pooled remission rate for psychological approaches compared to 49% for the combination. Dropout rates were higher for the combination compared to single psychological treatments.	Using a more conservative statistical approach, combination treatments were superior to single psychotherapy. This was the only statistically significant difference between treatments. The number of trials might be insufficient to show the statistical significance of a 19% absolute risk reduction in efficacy favouring psychotherapy or combination treatments over single antidepressants. Psychotherapy appeared to be more acceptable to subjects. When antidepressants were combined with psychological treatments, acceptability of the latter was significantly reduced.

INT-GSH: internet-based guided self-help; BIB-GSH: conventional guided bibliotherapy; BN: bulimia nervosa; ED: eating disorders; ESM training: emotional and social mind training; CBT: cognitive behavior therapy; ERP: exposure with response prevention; B-ERP: pre-binge exposure with response prevention; P-ERP: pre-purge exposure with response prevention; DSM-III-R: 3rd ed. of the Diagnostic and Statistical Manual of Mental Disorders; RCT: randomized controlled trials; DSM-IV: 4th ed. of the Diagnostic and Statistical Manual of Mental Disorders; DBT: dialectical behavior therapy; AF-DBT: appetite focused dialectical behavior therapy; iCBT: Internet-based cognitive-behavioural therapy; EDNOS: eating disorder not otherwise specified; WL/DTC: waiting list/delayed treatment control; CCP: client-centered psychotherapy; GSH: guided self help; FTF-CBT: face-to-face cognitive behavior therapy: TV-CBT: cognitive behavior therapy via telemedicine: EDE: Eating Disorder Examination; OR: *odds ratio*; FBT-BN: family-based treatment for bulimia nervosa; FBT: family-based treatment; SPT: supportive psychotherapy: FT: family therapy; SCL-90R: Symptom Checklist; EDI-2: Eating Disorder Inventory; AN: anorexia nervosa; CBT-BN: cognitive behavior therapy for bulimia nervosa; RTI-UNC EPC: RTI International-University of North Carolina at Chapel Hill Evidence-based Practice Center.

Extracted of PubMed

Table of findings - Binger eating disorder

Study	Objective/methods	Main results	Conclusions
de Zwaan M, Herpertz S, Zipfel S, Tuschen-Caffier B, Friederich HC, Schmidt F, et al. INTERBED: internet-based guided self-help for overweight and obese patients with full or subsyndromal binge eating disorder. A multicenter randomized controlled trial. Trials. 2012;21(13):220.	This pilot study will evaluate the efficacy of an INT-GSH program and CBT, which has been proven in several studies to be the gold standard treatment for BED, in a prospective multicenter randomized trial. Both treatments lasted 4 months, and maintenance of outcome will be assessed 6 and 18 months after the end of treatment. A total of 175 patients with BED and a BMI between 27 and 40kg/m^2 were randomized in 7 centers in Germany and Switzerland. Like most BED treatment trials, the difference in the number of binge eating days over the past 28 days is the primary outcome variable.	Although there is evidence that CBT is the first-line treatment for BED, it is not widely available. As BED is still a recent diagnostic category, many likely cases remain undiagnosed, and a large number of patients either receive delayed treatment or never get adequate treatment. A multicenter efficacy trial will give insight into the efficacy of a new INT-GSH program, allowing a direct comparison to the evidence-based gold standard treatment of CBT in German.	

continue...

...Continuation

Extracted of PubMed

Table of findings - Binger eating disorder

Study	Objective/methods	Main results	Conclusions
Grilo CM, Masheb RM, Crosby RD. Predictors and moderators of response to cognitive behavioral therapy and medication for the treatment of binge eating disorder. J Consult Clin Psychol. 2012;80(5):897-906.	To examine predictors and moderators of response to CBT and medication treatments for BED. Assessment of 108 BED patients in a randomized double-blind placebo-controlled trial, testing CBT and fluoxetine treatments, before, during and after therapy. Demographic factors, psychiatric and personality disorder comorbidity, ED psychopathology, psychological features, and two subtyping methods (negative affect, overvaluation of shape/weight) were tested as predictors and moderators for the primary outcome of remission from binge eating, and four secondary dimensional outcomes (binge-eating frequency, ED psychopathology, depression, and BMI).	One demographic variable signaled a statistical advantage for medication only (younger participants had greater binge-eating reductions), whereas several demographic and clinical variables (lower self-esteem, negative affect, and overvaluation of shape/weight) signaled better improvements if receiving CBT. Overvaluation was the most salient predictor/moderator of outcomes. Overvaluation significantly predicted binge-eating remission (29% of participants with *versus* 57% of participants without overvaluation remitted). Overvaluation was especially associated with lower remission rates if receiving medication only (10% *versus* 42% for participants without overvaluation). Overvaluation moderated dimensional outcomes: participants with overvaluation had significantly greater reductions in ED psychopathology and depression levels if receiving CBT. Overvaluation predictor/moderator findings persisted after controlling for negative affect.	Our findings have clinical utility for prescription of CBT and medication and implications for refinement of the BED diagnosis.
Robinson AH, Safer DL. Moderators of dialectical behavior therapy for binge eating disorder: results from a randomized controlled trial. Int J Eat Disord. 2012;45(4):597-602.	Investigate moderators of a randomized clinical trial of group DBT-BED compared to an ACGT on the post-treatment outcome of binge frequency after 20 two-hour weekly sessions. Moderation analyses. Participants were 101 adults with BED [mean (SD) age, 52.2 (10.6) years and BMI, 36.4 (8.6)].	Analyses identified two moderators of post-treatment outcome. Participants with (1) avoidant personality disorder or (2) an earlier onset of overweight and dieting (aged <15 years) showed significantly worsened outcome when treated with ACGT *versus* DBT-BED.	Participants with certain indicators of higher baseline pathology respond better to DBT-BED than ACGT at post-treatment.
Hilbert A, Bishop ME, Stein RI, Tanofsky-Kraff M, Swenson AK, Welch RR, et al. Long-term efficacy of psychological treatments for binge eating disorder. Br J Psychiatry. 2012;200(3):232-7.	To examine the long-term efficacy of outpatient CBT and IPT groups for BED and to analyse predictors of long-term non-response. Ninety people with BED were assessed 4 years after treatment cessation within a randomised trial.	Participants showed substantial long-term recovery, partial remission, clinically significant improvement and significant reductions in associated psychopathology, despite relapse tendencies in single secondary outcomes. BMI remained stable. While the IPT group demonstrated an improvement in ED symptoms. over the follow-up period, the CBT group reported a worsening of symptoms, but treatments did not differ at any time point.	The results document the long-term efficacy of out-patient CBT and IPT for BED. Further research is warranted to elucidate the time course and mechanisms of change of these treatments for BED.
Blomquist KK, Grilo CM. Predictive significance of changes in dietary restraint in obese patients with binge eating disorder during treatment. Int J Eat Disord. 2011;44(6):515-23.	To examine whether changes in different aspects of dietary restraint in obese patients with BED participating in a treatment study predict outcomes. Evaluation of 50 obese patients with BED in a RCT of Orlistat administered with CBT-GSH completed dietary restraint measures at baseline, during- and post-treatment, and three-month follow-up.	Change in the restraint scale of the EDE questionnaire did not predict binge abstinence or 5% weight loss. Increased flexible restraint subscale of the TFEQ during treatment significantly predicted binge abstinence at post-treatment and three-month follow-up and 5% weight loss at post-treatment. Change in the rigid restraint subscale of the TFEQ predicted binge abstinence at post-treatment.	Our findings clarify further pathologic and adaptive aspects of restraint and suggest the importance of enhancing flexible restraint in order to improve both binge eating and weight loss outcomes.
Carrard I, Crépin C, Rouget P, Lam T, Golay A, Van der Linden M. Randomised controlled trial of a guided self-help treatment on the Internet for binge eating disorder. Behav Res Ther. 2011;49(8):482-91.	To evaluate the efficacy of an INT-GSH treatment programme, based on CBT, for adults with threshold and subthreshold BED. Randomization of 74 women into 2 groups. The first group received the 6-month on-line programme with a 6-month follow-up. The second group was placed in a 6-month waiting list before participating in the 6-month intervention. Guidance consisted of a regular e-mail contact with a coach during the whole intervention.	Binge eating behaviour, drive for thinness, body dissatisfaction and interoceptive awareness significantly improved after the internet self-help treatment intervention. The number of objective binge episodes, overall ED symptoms score and perceived hunger also decreased. Improvements were maintained at six-month follow-up. Dropouts exhibited more shape concern and a higher drive for thinness.	Overall, a transfer of CBT-based self-help techniques to the Internet was well accepted by patients, and showed positive results for ED psychopathology.

continue...

...Continuation

Extracted of PubMed

Table of findings - Binger eating disorder

Study	Objective/methods	Main results	Conclusions
Castelnuovo G, Manzoni GM, Villa V, Cesa GL, Pietrabissa G, Molinari E. The STRATOB study: design of a randomized controlled clinical trial of Cognitive Behavioral Therapy and Brief Strategic Therapy with telecare in patients with obesity and binge-eating disorder referred to residential nutritional rehabilitation. Trials. 2011;12:114.	The comparison between CBT and BST will be assessed in a two-arm RCT. Due to the novelty of the application of BST in BED treatment, this pilot study will be carried out before conducting a large scale RCT. Both CBT and BST group will follow an in-hospital treatment (diet, physical activity, dietitian counseling, and eight psychological sessions) plus eight out-patient telephone-based sessions of psychological support and monitoring with the same in-patient psychotherapists.	Primary outcome measure of the randomized trial will be the change in the OQ-45.2. Secondary outcome measures will be the percentage of BED patients remitted considering the number of weekly binge episodes and the weight loss. Data will be collected at baseline, at discharge from the hospital (c.a. 1 month after) and after 6, 12 and 24 months from the end of the in-hospital treatment. Data at follow-up time points will be collected through tele-sessions.	The STRATOB (Systemic and STRATegic psychotherapy for Obesity), a comprehensive two-phase stepped down program enhanced by telepsychology for the medium-term treatment of obese people with BED seeking intervention for weight loss, will shed light about the comparison of the effectiveness of the BST with the gold standard CBT and about the continuity of care at home using a low-level of telecare (mobile phones).
Safer DL, Joyce EE. Does rapid response to two group psychotherapies for binge eating disorder predict abstinence? Behav Res Ther. 2011;49(5):339-45.	Extend understanding of a RR to treatment by examining its prognostic significance at end-of-treatment and 1 year follow-up within two group treatments for BED: DBT-BED and an active comparison group therapy. A total of 101 adults with BED randomized to 20-weeks DBT-BED *versus* active comparison group therapy. RR defined as ≥65% reduction in the frequency of days of binge eating by week 4.	(1) Significantly higher binge eating abstinence for rapid responders (n=41) *versus* non-rapid responders (n=60) at EOT (70.7% *versus* 33.3%) and 1-year follow-up (70.7% *versus* 40.0%), respectively, as well as improvement on most secondary measures. (2) Significantly less attrition among RR *versus* non-RR. (3) Significantly higher binge eating abstinence rates at both time points for DBT-RR *versus* DBT-non-RR, but not for active comparison group therapy-RR *versus* active comparison group therapy.-non-RR.	Current study extends prognostic significance of RR to 1-year follow-up. RR more prominent for those randomly assigned to DBT-BED than active comparison group therapy.
Striegel-Moore RH, Wilson GT, DeBar L, Perrin N, Lynch F, Rosselli F, Kraemer HC. Cognitive behavioral guided self-help for the treatment of recurrent binge eating. J Consult Clin Psychol. 2010;78(3):312-21.	This efficacy-effectiveness study sought to evaluate whether a manual-based CBT-GSH, delivered in eight sessions in a health maintenance organization setting over a 12-week period by master's-level interventionists, is more effective than treatment as usual. Randomization of 123 individuals (mean age = 37.2; 91.9% female, 96.7% non-Hispanic White), including 10.6% with BN, 48% with BED, and 41.4% with recurrent binge eating in the absence of BN or BED.	At 12-month follow-up, CBT-GSH resulted in greater abstinence from binge eating (64.2%) than treatment as usual (44.6%; number needed to treat = 5), as measured by the EDE. Secondary outcomes reflected greater improvements in the CBT-GSH group in dietary restraint (d=0.30); eating, shape, and weight concern (ds=0.54, 1.01, 0.49, respectively; measured by the EDE questionnaire); depression (d=0.56; Beck Depression Inventory); and social adjustment (d=0.58; Work and Social Adjustment Scale), but not weight change.	CBT-GSH is a viable first-line treatment option for the majority of patients with recurrent binge eating who do not meet diagnostic criteria for BN or AN.
Ricca V, Castellini G, Mannucci E, Lo Sauro C, Ravaldi C, Rotella CM, et al. Comparison of individual and group cognitive behavioral therapy for binge eating disorder. A randomized, three-year follow-up study. Appetite. 2010;55(3):656-65.	To evaluate the effectiveness of individual and group CBT, and the possible predictors of outcome. At the beginning, at the end of treatment, and 3 years after the end of treatment, 144 patients affected by threshold or subthreshold BED were assessed. The following outcome measures were considered: recovery at 3-year follow-up, weight loss, treatment resistance, relapse, and diagnostic change.	Both treatments showed similar response in terms of all outcome measures in the long-term, and determined a significant reduction of binge eating frequency, and a mild reduction of weight. The absence of a history of amphetamine derivatives consumption, lower emotional eating and binge eating severity at baseline were predictors of full recovery in the long-term. A low emotional eating was found to be the only predictor of weight reduction. Overweight during childhood, full blown BED diagnosis, and high emotional eating were predictors of treatment resistance.	Treatments considering the relation between binge eating and emotional eating could improve the outcome of BED patients.
Safer DL, Robinson AH, Jo B. Outcome from a randomized controlled trial of group therapy for binge eating disorder: comparing dialectical behavior therapy adapted for binge eating to an active comparison group therapy. Behav Ther. 2010;41(1):106-20. Erratum in: Behav Ther. 2010;41(3):432. Robinson, Athena Hasler [added].	DBT-BED aims to reduce binge eating by improving adaptive emotion-regulation skills. To control for the hypothesized specific effects of DBT-BED, the present study compared DBT-BED to an ACGT. Men and women (n=101) meeting DSM-IV BED research criteria were randomly assigned to 20 group sessions of DBT-BED (n=50) or ACGT (n=51).	DBT-BED had a significantly lower dropout rate (4%) than ACGT (33.3%). Linear mixed models revealed that post-treatment binge abstinence and reductions in binge frequency were achieved more quickly for DBT-BED than for ACGT (post-treatment abstinence rate=64% for DBT-BED *versus* 36% for ACGT) though differences did not persist over the 3, 6, and 12-month follow-up assessments (*e.g.*, 12-month follow-up abstinence rate of 64% for DBT-BED *versus* 56% for ACGT). Secondary outcome measures revealed no sustained impact on emotion regulation.	Although both DBT-BED and ACGT reduced binge eating, DBT-BED showed significantly fewer dropouts and greater initial efficacy (*e.g.*, at post-treatment) than ACGT. The lack of differential findings over follow-up suggests that the hypothesized specific effects of DBT-BED do not show long-term impact beyond those attributable to nonspecific common therapeutic factors.

continue...

...Continuation

Extracted of PubMed

Table of findings - Binger eating disorder

Study	Objective/methods	Main results	Conclusions
Lynch FL, Striegel-Moore RH, Dickerson JF, Perrin N, Debar L, Wilson GT, et al. Cost-effectiveness of guided self-help treatment for recurrent binge eating. J Consult Clin Psychol. 2010;78(3):322-33.	To conduct an incremental CEA of a CBT-GSH to treat recurrent binge eating compared to treatment as usual. Participants were 123 adult members of an HMO (mean age of 37.2 years, 91.9% female, 96.7% non-Hispanic White) who met criteria for ED involving binge eating as measured by the EDE. Participants were randomized either to treatment as usual or to treatment as usual plus CBT-GSH. The clinical outcomes were binge-free days and (QALYs); total societal cost was estimated using costs to patients and the health plan and related costs.	Compared to those receiving treatment as usual only, those who received treatment as usual plus CBT-GSH experienced 25.2 more binge-free days and had lower total societal costs of US$ 427 over 12 months following the intervention (incremental CEA ratio of - US$ 20.23 per binge-free day or - US$ 26,847 per QALY). Lower costs in the treatment as usual plus CBT-GSH group were due to reduced use of treatment as usual services in that group, resulting in lower net costs for the treatment as usual plus CBT group despite the additional cost of CBT-GSH.	Findings support CBT-GSH dissemination for recurrent binge-eating treatment.
Wilson GT, Wilfley DE, Agras WS, Bryson SW. Psychological treatments of binge eating disorder. Arch Gen Psychiatry. 2010;67(1):94-101.	To test whether patients with BED require specialty therapy beyond behavioral weight loss treatment and whether IPT is more effective than either behavioral weight loss treatment or CBT-GSH in patients with a high negative affect during a 2-year follow-up. A total of 205 women and men with a BMI between 27 and 45 who met DSM-IV criteria for BED. Intervention Twenty sessions of IPT or behavioral weight loss treatment or 10 sessions of CBT-GSH during 6 months.	At 2-year follow-up, both IPT and CBT-GSH resulted in greater remission from binge eating than behavioral weight loss treatment ($p<0.05$; odds ratio: behavioral weight loss treatment *versus* CBT-GSH, 2.3; behavioral weight loss treatment *versus* IPT, 2.6; and CBT-GSH *versus* IPT, 1.2). self-esteem ($p<0.05$) and global EDE ($p<0.05$) scores were moderators of treatment outcome. The odds ratios for low and high global EDE scores were 2.8 for behavioral weight loss treatment, 2.9 for CBT-GSH, and 0.73 for IPT; for self-esteem, they were 2.4 for behavioral weight loss treatment, 1.9 for CBT-GSH, and 0.9 for IPT.	IPT and CBT-GSH are significantly more effective than behavioral weight loss treatment in eliminating binge eating after 2 years. Guided self-help based on cognitive behavior therapy is a first-line treatment option for most patients with BED, with IPT (or full cognitive behavior therapy) used for patients with low self-esteem and high ED psychopathology.
Peterson CB, Mitchell JE, Crow SJ, Crosby RD, Wonderlich SA. The efficacy of self-help group treatment and therapist-led group treatment for binge eating disorder. Am J Psychiatry. 2009;166(12):1347-54.	To compare three types of treatment for BED and determine the relative efficacy of Self-help group treatment compared to therapist-led and therapist-assisted group CBT. A total of 259 adults diagnosed with BED were randomly assigned to 20 weeks of therapist-led, therapist-assisted, or self-help group treatment or a waiting list condition. Binge eating as measured by the EDE was assessed at baseline, at end of treatment, and at 6 and 12 months, and outcome was assessed using logistic regression and analysis of covariance (intent-to-treat).	At end of treatment, the therapist-led (51.7%) and the therapist-assisted (33.3%) conditions had higher binge eating abstinence rates than the self-help (17.9%) and waiting list (10.1%) conditions. However, no differences in abstinence rates were observed among groups at any follow-up assessments. The therapist-led condition also showed more reductions in binge eating at end of treatment and follow-up assessments compared to the self-help condition, and treatment or waiting period completion rates were higher in the therapist-led (88.3%) and waiting list (81.2%) conditions than in the therapist-assisted (68.3%) and self-help (59.7%) conditions.	Therapist-led group CBT for BED led to higher binge eating abstinence rates, greater reductions in binge eating frequency, and lower attrition compared to group self-help treatment. Although these findings indicate that therapist delivery of group treatment is associated with better short-term outcome and less attrition than self-help treatment, the lack of group differences at follow-up suggests that self-help group treatment may be a viable alternative to therapist-led interventions.
Brambilla F, Samek L, Company M, Lovo F, Cioni L, Mellado C. Multivariate therapeutic approach to binge-eating disorder: combined nutritional, psychological and pharmacological treatment. Int Clin Psychopharmacol. 2009;24(6):312-7.	In 30 BED patients, we monitored the effects of three types of 6-month treatment, randomly assigned to one of the three treatment groups, each consisting of 10 patients. Group 1 received a 1,700-kcal diet (21% proteins, 27% lipids, 52% carbohydrate), CBT, sertraline (50-150 mg/day) and topiramate (25-150 mg/day); Group 2 received the same diet, CBT, sertraline; and group 3 received nutritional counselling and CBT. Binge frequency and weight were assessed every month.	Binge frequency and excessive weight decreased significantly only in Group 1 patients, in whom improvement was noted in total EDI scores and the subitems 'bulimia', 'drive for thinness', 'maturity fear', 'ascetism', in total SCL-90-R scores and in the subitem 'somatization', in PDQ-4-R subitems 'schizotypic personality' and 'dependent personality'. Group 2 patients improved on the SCL-90-R subitems 'depression' and 'interpersonal relationship' and in the PDQ-4-R 'schizoid personality'.	Combination therapy seems to be the only fully effective treatment in BED patients.

continue...

...Continuation

Extracted of PubMed

Table of findings - Binger eating disorder

Study	Objective/methods	Main results	Conclusions
Cassin SE, von Ranson KM, Heng K, Brar J, Wojtowicz AE. Adapted motivational interviewing for women with binge eating disorder: a randomized controlled trial. Psychol Addict Behav. 2008;22(3):417-25.	In this RCT, 108 women with BED recruited from the community were assigned to either an adapted motivational interviewing group (1 individual adapted motivational interviewing session + self-help handbook) or control group (handbook only). They were called over the phone at 4, 8, and 16 weeks following the initial session to assess binge eating and associated symptoms (depression, self-esteem, quality of life).	After intervention, the adapted motivational interviewing group participants were more confident than those in the control group in their ability to change binge eating. Although both groups reported improved binge eating, mood, self-esteem, and general quality of life 16 weeks following the intervention, the adapted motivational interviewing group improved to a greater extent. A greater proportion of women in the adapted motivational interviewing group abstained from binge eating (27.8% *versus* 11.1%) and no longer met the binge frequency criterion of the DSM IV TR for BED (87.0% *versus* 57.4%).	Adapted motivational interviewing may constitute a brief, effective intervention for BED and associated symptoms.
Grilo CM, Masheb RM. Rapid response predicts binge eating and weight loss in binge eating disorder: findings from a controlled trial of orlistat with guided self-help cognitive behavioral therapy. Behav Res Ther. 2007;45(11):2537-50.	We examined RR in obese patients with BED who participated in a randomized placebo-controlled study of Orlistat administered with CBT-GSH format. 50 patients were randomly assigned to 12-week treatments of either Orlistat + CBT-GSH or placebo + CBT-GSH, and were followed in double-blind fashion for 3 months after treatment discontinuation. RR, defined as 70% or greater reduction in binge eating by the fourth treatment week, was determined by receiver operating characteristic curves, and was then used to predict outcomes.	RR characterized 42% of participants, was unrelated to participants' demographic features and most baseline characteristics, and was unrelated to attrition from treatment. Participants with RR were more likely to achieve binge eating remission and 5% weight loss. If RR occurred, the level of improvement was sustained during the remaining course of treatment and the 3-month period after treatment. Participants without RR showed a subsequent pattern of continued improvement.	RR demonstrated the same prognostic significance and time course for CBT-GSH as previously documented for individual CBT. Among rapid responders, improvements were well sustained, and among non-rapid responders, continuing with CBT-GSH (regardless of medication) led to subsequent improvements.
Shelley-Ummenhofer J, MacMillan PD. Cognitive-behavioural treatment for women who binge eat. Can J Diet Pract Res. 2007;68(3):139-42.	A dietitian-administered, shortened form of the Apple and Agras CBT method was evaluated in a group setting to determine its effect on improving obese women's self-esteem and reducing binge-eating behaviours, depression, and negative body image. Respondents who met study selection criteria were randomly assigned to either a CBT group (n=13) or a delayed CBT group (n=9). The treatment was administered over 6 weekly sessions to the CBT group, and then twice weekly over three weeks to the delayed CBT group.	The intervention did not result in any changes in body weight. There were statistically significant and clinically important changes after treatment ($p<0.05$) for all five measures. Binge-eating severity and frequency decreased, depression decreased, body image improved, and self-esteem improved. All changes were greater in the 6-week treatment group.	The dietitian-administered, group setting CBT program is effective for reducing binge eating and improving emotional state in obese women.
Claudino AM, de Oliveira IR, Appolinario JC, Cordás TA, Duchesne M, Sichieri R, et al. Double-blind, randomized, placebo-controlled trial of topiramate plus cognitive-behavior therapy in binge-eating disorder. J Clin Psychiatry. 2007;68(9):1324-32.	To evaluate the efficacy and tolerability of adjunctive topiramate compared to placebo in reducing weight and binge eating in obese patients with BED receiving CBT. A double-blind, randomized, placebo-controlled trial of 21 weeks' duration was conducted at four university centers. Participants were 73 obese (BMI $\geq$30 kg/m^2) outpatients with BED (DSM-IV criteria), both genders, and aged from 18 to 60 years. After a 2- to 5-week run-in period, selected participants were treated with group CBT (19 sessions) and topiramate (target daily dose of 200. mg) or placebo (September 2003 to April 2005).	Repeated-measures random regression analysis revealed a greater rate of weight reduction associated with topiramate over the course of treatment ($p<0.001$), with patients taking topiramate attaining a clinically significant weight loss (-6.8kg) compared to patients taking placebo (-0.9kg). Although rates of reduction of binge frequencies, BES scores, and BDI scores did not differ between groups during treatment, a greater number of patients of the Topiramate plus CBT group (31/37) attained binge remission compared to patients taking placebo (22/36) during the trial ($p=0.03$). No difference between groups was found in completion rates; one patient (topiramate group) withdrew for adverse effect.	Topiramate added to CBT improved the efficacy of the later, increasing binge remission and weight loss in the short run. Topiramate was well tolerated, as shown by few adverse events during treatment.

continue...

...Continuation

Extracted of PubMed

Table of findings - Binger eating disorder

Study	Objective/methods	Main results	Conclusions
Hilbert A, Saelens BE, Stein RI, Mockus DS, Welch RR, Matt GE, et al. Pretreatment and process predictors of outcome in interpersonal and cognitive behavioral psychotherapy for binge eating disorder. J Consult Clin Psychol. 2007;75(4):645-51.	To examine pretreatment and process predictors of individual nonresponse to psychological group treatment of BED. In a randomized trial, 162 overweight patients with BED were treated with either group CBT or group IPT. Treatment nonresponse, which was defined as nonabstinence from binge eating, was assessed at post-treatment. and at one year following treatment completion.	Using four signal detection analyses, greater extent of interpersonal problems prior to treatment or at midtreatment were identified as predictors of nonresponse, both at post-treatment and at 1-year follow-up. Greater pretreatment and midtreatment concerns about shape and weight, among those patients with low interpersonal problems, were predictive of post-treatment nonresponse. Lower group cohesion during the early treatment phase predicted nonresponse at one-year follow-up.	Attention to specific pre- or in-treatment predictors could allow for targeted selection into differential or augmented care and could thus improve response to group psychotherapy for BED.
Shapiro JR, Reba-Harrelson L, Dymek-Valentine M, Woolson SL, Hamer RM, Bulik CM. Feasibility and acceptability of CD-ROM-based cognitive-behavioural treatment for binge-eating disorder. Eur Eat Disord Rev. 2007;15(3):175-84.	To compare preliminary feasibility and acceptability of CD-ROM-delivered CBT for overweight individuals with BED to 10 weekly group CBT sessions and to a waiting list control.	Attrition was numerically greater in the group CBT sessions than the CD-ROM condition; although only group CBT sessions differed significantly from waiting list control in dropout rates. Those in the CD-ROM condition reported continued use of their CD-ROM after treatment. Also, the majority of waiting list control participants elected to receive CD-ROM over group CBT sessions treatment at the end of the waiting period. Preliminarily, no significant differences emerged across the active treatment groups on most outcome measures. However, there was a significantly greater decline in binge days in the two active groups relative to waiting list control.	CD-ROM appears to be an acceptable and at least initially preferred method of CBT delivery for overweight individuals with BED.
Ljotsson B, Lundin C, Mitsell K, Carlbring P, Ramklint M, Ghaderi A. Remote treatment of bulimia nervosa and binge eating disorder: a randomized trial of Internet-assisted cognitive behavioural therapy. Behav Res Ther. 2007;45(4):649-61.	The present study investigated the efficacy of self-help based on CBT in combination with internet support in the treatment of BN and BED. After confirming the diagnosis with an in-person interview, 73 patients were randomly allocated to treatment or a waiting list control group.	Treated individuals showed marked improvement after 12 weeks of Self-help compared to the control group on both primary and secondary outcome measures. Intent-to-treat analyses revealed that 37% (46% among completers) had no binge eating or purging at the end of the treatment and a considerable number of patients achieved clinically significant improvement on most of the other measures as well.	The results were maintained at 6-month follow-up, and provide evidence to support the continued use and development of self-help programmes.
Munsch S, Biedert E, Meyer A, Michael T, Schlup B, Tuch A, et al. A randomized comparison of cognitive behavioral therapy and behavioral weight loss treatment for overweight individuals with binge eating disorder. Int J Eat Disord. 2007;40(2):102-13.	To determine the efficacy of CB) and behavioral weight loss treatment (BWLT) for overweight patients with BED. Eighty obese patients meeting criteria of BED according to DSM-IV TR were randomly assigned to either CBT or behavioral weight loss treatment, consisting of 16 weekly treatments and 6 monthly follow-up sessions. Binge eating, general psychopathology, and BMI were assessed before, during, and after treatment, and at 12-month follow-up.	At post-treatment results favored CBT as the more effective therapy. Analysis of the course of treatments pointed to a faster improvement of binge eating in CBT based on the number of self-reported weekly binges, but faster reduction of BMI in behavioral weight loss treatment. At 12-month follow-up, no substantial differences between the two treatment conditions existed.	CBT was somewhat more efficacious than behavioral weight loss treatment in treating binge eating but this superior effect was barely maintained in the long term. Further research into cost effectiveness is needed to assess which treatment should be considered the treatment of choice.
Grilo CM, Masheb RM, Wilson GT. Rapid response to treatment for binge eating disorder. Consult Clin Psychol. 2006;74(3):602-13.	The authors examined RR among 108 patients with BED who were randomly assigned to one of four 16-week treatments: fluoxetine, placebo, CBT plus fluoxetine, or CBT plus placebo. RR, defined as 65% or greater reduction in binge eating by the fourth treatment week, was determined by receiver operating characteristic curves. RR characterized 44% of participants and was unrelated to participants' demographic or baseline characteristics.	Participants with RR were more likely to achieve binge-eating remission, had greater improvements in eating-disorder psychopathology, and had greater weight loss than participants without RR. RR had different prognostic significance and distinct time courses for CBT *versus* pharmacotherapy-only treatments.	RR has utility for predicting outcomes and provides evidence for specificity of treatment effects with BED.

continue...

...Continuation

Extracted of PubMed

Table of findings - Binger eating disorder

Study	Objective/methods	Main results	Conclusions
Dunn EC, Neighbors C, Larimer ME. Motivational enhancement therapy and self-help treatment for binge eaters. Psychol Addict Behav. 2006;20(1):44-52.	To evaluate whether a single session of MET would increase participant readiness to change, improve the efficacy of self-help treatment for binge eaters, and improve participant compliance with the self-help manual. Participants with bulimia nervosa or BED were randomly assigned either to attend a 1-hour MET session prior to receiving the self-help manual (n=45) or to receive the self-help manual only (n=45).	The MET intervention resulted in increased readiness to change for binge eating compared with the self-help-only condition. Few differences were found between the MET condition and the self-help-only condition for changes in eating attitudes and frequency of binge eating and compensatory behaviors. No significant effects were found for compliance.	This research adds to the literature regarding the use of brief motivational interventions to enhance readiness for change in populations with ED.
Molinari E, Baruffi M, Croci M, Marchi S, Petroni ML. Binge eating disorder in obesity: comparison of different therapeutic strategies. Eat Weight Disord. 2005;10(3):154-61.	To compare different integrated therapeutic approaches for the BED. A sample of 65 female severely obese BED was randomly divided into three groups: the first one was treated by CBT alone; the second one was treated by SSRI antidepressant therapy (fluoxetine) alone; the remaining was treated by a combination of CBT plus fluoxetine. All groups received group nutritional training and individual dietary counselling. The initial fluoxetine dose (20mg/day) was adjusted (up to 60mg/day) according to frequency of binge eating.	During the first 4 weeks, all subjects underwent an in-patient dietary treatment aimed to achieve at least a 5% weight loss, which was continued during the outpatient treatment phase. The results showed the two groups that underwent psychotherapy resulted in a better outcome - in terms of number of bingeing episodes, maintenance of weight loss reduction from baseline and psychological well being - than the group treated with pharmacological therapy alone.	The study underlines the importance of a multidisciplinary approach to the treatment of BED.
**Systematic review/meta-analysis**	**Objective/methods**	**Main results**	**Conclusions**
Palavras MA, Kaio GH, Mari JJ, Claudino AM. Uma revisão dos estudos latino-americanos sobre o transtorno da compulsão alimentar periódica. Rev Bras Psiquiatr. 2011;33 Suppl 1:S81-108. Review.	To review the state of the art of the scientific literature on BED in Latin America. A literature search of studies conducted in Latin American countries using the term “binge eating” was performed in the following electronic databases: PubMed, LILACS, SciELO, and PsycINFO. Selected articles described studies developed with Latin American samples that met partial or complete DSM-IV diagnostic criteria for BED.	30 studies met the inclusion criteria (18 cross-sectional studies, 5 clinical trials, 4 case reports, 2 validity studies, and 1 cohort study). Most of the studies were conducted in Brazil (27), one in Argentina, one in Colombia, and one in Venezuela. The prevalence of BED among obese people attending weight loss programs ranged between 16% and 51.6%. The comparison between obese people with and without BED showed a tendency of higher weight, longer history of weight fluctuation, more concern about shape and weight, and association with psychiatric comorbidity in those with BED.	BED is a detectable phenomenon in Latin America with clinical features similar to those found in the international literature. This review provides support for the consideration of BED as a distinct ED in the International Classification of Diseases - 11th edition.
Vocks S, Tuschen-Caffier B, Pietrowsky R, Rustenbach SJ, Kersting A, Herpertz S. Meta-analysis of the effectiveness of psychological and pharmacological treatments for binge eating disorder. Int J Eat Disord. 2010;43(3):205-17. Review.	To compute and compare mean effects of various treatments for BED. Thirty-eigth studies with 1,973 participants fulfilled the defined inclusion criteria. Effect sizes, odds ratios, and simple rates were integrated in fixed and random (mixed) effects categorical models.	Psychotherapy and structured self-help, both based on cognitive behavioral interventions, were found to have large effects on the reduction of binge eating. Regarding pharmacotherapy, mainly comprising antidepressants, RCT revealed medium effects for the reduction of binge eating. Uncontrolled studies on weight-loss treatments demonstrated moderate reductions of binge eating. Combination treatments did not result in higher effects compared with single-treatment regimens. Except for weight-loss treatment, none of the interventions resulted in a considerable weight reduction.	Psychotherapy and structured self-help, both based on cognitive-behavioral interventions, should be recommended as the first-line treatments.

continue...

...Continuation

Extracted of PubMed

Table of findings - Binger eating disorder

Systematic review/meta-analysis	Objective/methods	Main results	Conclusions
Brownley KA, Berkman ND, Sedway JA, Lohr KN, Bulik CM. Binge eating disorder treatment: a systematic review of randomized controlled trials. Int J Eat Disord. 2007;40(4):337-48. Review.	The RTI-UNC EPC systematically reviewed evidence on efficacy of treatment for BED, harms associated with treatments, factors associated with treatment efficacy, and differential outcome by sociodemographic characteristics. A total of 26 studies, including medication-only, medication plus behavioral intervention, and behavioral intervention only designs, met inclusion criteria.	The strength of the evidence for medication and behavioral interventions was moderate, for self-help and other interventions was weak, for treatment-related harms was strong, for factors associated with efficacy of treatment was weak, and for differential outcome by sociodemographic factors was nonexistent. Individual or group CBT reduces binge eating and improves abstinence rates for up to 4 months after treatment but does not lead to weight loss. Medications may play a role in treating BED patients.	The literature regarding treatment efficacy for BED is variable. Future directions include the identification of optimal interventions that are associated with both sustained abstinence from binge eating and permanent weight loss.

INT-GSH: internet-based guided self-help; CBT: cognitive behavior therapy; BED: binge eating disorder; BMI: body mass index; ED: eating disorder; DBT-BED: dialectical behavior therapy for binge eating disorder; ACGT: active Comparison group control; SD: standard deviation; IPT: interpersonal psychotherapy; CBT-GSH: cognitive-behavioral therapy, guided-self-help; EDE: Eating Disorder Examination; TFEQ: *Three Factor Eating Questionnaire*; BST: brief strategic therapy; RCT: randomized controlled clinical trial; OQ-45.2: Global Index of the Outcome Questionnaire; RR: rapid response; CBT-GSH: cognitive behavior therapy based guided self-help; BN: bulimia nervosa; AN: anorexia nervosa; DSM-IV: 4th ed. of the Diagnostic and Statistical Manual of Mental Disorders; CEA: cost-effectiveness analysis; QALYs: quality-adjusted life years; IPT: interpersonal psychotherapy; EDI: *Eating Disorder Inventory-2;* SCL-90-R: *Symptom Checklist-90-Revised*; PDQ-4-R: Personality Diagnostic Questionnaire-4+; DSM IV TR:; BES:; BDI:; MET: motivational enhancement therapy; SSRI: selective serotonin reuptake inhibitor; RTI-UNC EPC: RTI International-University of North Carolina at Chapel Hill Evidence-based Practice Center.

Extracted of PubMed

Table of findings - Eating disorders

Study	Objective/methods	Main results	Conclusions
Stein KF, Corte C, Chen DG, Nuliyalu U, Wing J. A randomized clinical trial of an identity intervention programme for women with eating disorders. Eur Eat Disord Rev. 2013;21(2):130-42.	Findings of a randomized trial of an IIP designed to build new positive self-schemas that are separate from other conceptions of the self in memory as the means to promote improved health in women diagnosed with ED are reported. After baseline data collection, women with AN or BN were randomly assigned to IIP (n=34) or SPI (n=35).	The IIP and SPI were equally effective in reducing ED symptoms at 1-month post-intervention, and changes were stable through the 12-month follow-up period. The IIP tended to be more effective in fostering development of positive self-schemas, and the increase was stable over time. Regardless of baseline level, an increase in the number of positive self-schemas between pre-intervention and one-month post-intervention predicted a decrease in desire for thinness and an increase in psychological well-being and functional health over the same period.	A cognitive behavioural intervention that focuses on increasing the number of positive self-schemas may be central to improving emotional health in women with AN and BN.
ter Huurne ED, Postel MG, de Haan HA, Drossaert CH, DeJong CA. Web-based treatment program using intensive therapeutic contact for patients with eating disorders: before-after study. J Med Internet Res. 2013;15(2):e12.	This pilot study evaluated the web-based treatment program using intensive therapeutic contact in a population of 165 patients with an ED. In a pre-post design with 6-week and 6-month follow-ups, ED psychopathology, body dissatisfaction, BMI, physical and mental health, and quality of life were measured. The participant's satisfaction with the web-based treatment program was also studied. Attrition data were collected, and participants were classified as noncompleters if they did not complete all ten assignments of the web-based treatment program. Differences in treatment effectiveness, treatment adherence, and baseline characteristics between participants of the three major ED diagnostic groups EDNOS (n=115), BN purging (n=24), and BN nonpurging (n=24) were measured.	Of the 165 participants who started the web-based treatment program, 89 participants (54%) completed all of the program assignments (completers) and 76 participants (46%) ended the program prematurely (noncompleters). Severe body dissatisfaction and physical and mental health problems seemed to have a negative impact on the completion of the web-based treatment program. Among the participants who completed the treatment program, significant improvements were found in ED psychopathology ($F=54.6$; $df=68$; $p<0.001$; $d=1.14$). Body dissatisfaction, quality of life, and physical and mental health also significantly improved, and almost all of these positive effects were sustained up to 6 months after the participants had completed the web-based treatment program. BMI improved only within the group of participants suffering from obesity. The improvement in ED psychopathology occurred in all three ED diagnostic groups, and the percentage of completers did not differ significantly between these groups. Participants' satisfaction with the treatment program, as well as with their therapist, was high, and participants indicated that they would recommend the program to other patients with ED.	The results of this study suggest that the web-based treatment program has the potential to improve ED psychopathology in patients with different types of ED.

continue...

...Continuation

Extracted of PubMed

Table of findings - Eating disorders

Study	Objective/methods	Main results	Conclusions
Bhatnagar KA, Wisniewski L, Solomon M, Heinberg L. Effectiveness and feasibility of a cognitive-behavioral group intervention for body image disturbance in women with eating disorders. J Clin Psychol. 2013;69(1):1-13.	To investigate the effectiveness and feasibility of a cognitive-behavioral group intervention for the treatment of body image disturbance in women with ED. The study used a multiple-baseline design and enrolled 38 participants with a range of ED. The intervention targeted attitudinal and behavioral components of body image disturbance using psychoeducation, self-monitoring, systematic desensitization, and cognitive restructuring. Primary outcomes included multidimensional body image assessment (effectiveness) and treatment adherence and satisfaction (feasibility).	Participants undergoing manualized Group treatment reported significantly less body image disturbance than participants randomized to a Waitlist control condition. However, differences disappeared after both groups had been through intervention.Participants also reported less depression and ED pathology from baseline to post-treatment, however this difference was not considered statistically significant. Feasibility outcomes suggest the intervention was well received and highly acceptable to participants.	Findings emphasize the importance of adding an evidence-based body image component to standard eating ED.
Courbasson C, Nishikawa Y, Dixon L. Outcome of dialectical behaviour therapy for concurrent eating and substance use disorders. Clin Psychol Psychother. 2012;19(5):434-49.	To examine the preliminary efficacy of DBT adapted for concurrent ED and SUD. A matched RCT was carried out with 25 female outpatients diagnosed with concurrent ED and SUD. Participants randomized to the intervention condition received DBT, whereas those randomized to the control condition received treatment as usual, both for a period of one year.	Participants randomized to the DBT condition evidenced a superior retention rate relative to their counterparts in the treatment as usual condition at various study time points, including post-treatment (80% *versus* 20%) and follow-up (60% *versus* 20%). Due to the unexpected elevated dropout rates and the worsening of ED-SUD symptomatology in the treatment as usual condition, recruitment efforts were terminated early. Results from the DBT condition revealed that the intervention had a significant positive effect on behavioural and attitudinal features of disordered eating, substance use severity and use, negative mood regulation and depressive symptoms. Finally, increases in participants' perceived ability to regulate and cope with negative emotional states were significantly associated with decreases in emotional eating and increases in levels of confidence in ability to resist urges for substance use.	Results suggest that the adapted DBT might hold promise for treating individuals with concurrent ED and SUD. The results bear upon the highly salient and important issue of whether individuals with concurrent substance use need to be excluded from research studies and treatment programmes.
Bauer S, Okon E, Meermann R, Kordy H. Technology-enhanced maintenance of treatment gains in eating disorders: efficacy of an intervention delivered via text messaging. J Consult Clin Psychol. 2012;80(4):700-6.	Given the lack of maintenance interventions for ED, a program delivered via the short message service (SMS) and text messaging was developed to support patients after their discharge from inpatient treatment. A total of 165 female patients with BN or a related EDNOS were randomly assigned to a control group (treatment as usual) or an intervention group (SMS-based maintenance intervention; SMS). After hospital discharge, participants in the intervention group submitted a weekly symptom report via text message for 16 weeks and received tailored feedback. Primary outcome was the rate of partial remission 8 months after discharge from inpatient treatment.	The difference in remission rates reached significance in the intent-to-treat analyses (SMS =51.2%; treatment as usual =36.1%), $\chi^2(1)=3.81$; p=0.05, and approached significance in the completer analysis (SMS=59.2%; treatment as usual =43.5%), $\chi^2(1)=3.44$; p=0.06. There were no differences in the utilization of outpatient treatment. Remission rates between the intervention and control groups were not significantly different among patients who used outpatient treatment (63.2% *versus* 55.6%), $\chi^2(1)=0.44$, p=0.51. A significant difference was found in those who did not utilize such treatment (54.5% *versus* 30.3%), $\chi^2(1)=3.97$; p=0.046.	The aftercare intervention was efficacious in enhancing treatment outcome after discharge from inpatient treatment.
Alberts HJ, Thewissen R, Raes L. Dealing with problematic eating behaviour. The effects of a mindfulness-based intervention on eating behaviour, food cravings, dichotomous thinking and body image concern. Appetite. 2012;58(3):847-51.	To explore the efficacy of a mindfulness-based intervention for problematic eating behavior. A non-clinical sample of 26 women with disordered eating behavior was randomly assigned to an 8-week MBCT-based eating intervention or a Waiting list control group. Data were collected at baseline and after 8 weeks.	Compared to controls, participants in the Mindfulness Intervention showed significantly greater decreases in food cravings, dichotomous thinking, body image concern, emotional eating and external eating.	These findings suggest that mindfulness practice can be an effective way to reduce factors that are associated with problematic eating behavior.

continue...

...Continuation

Extracted of PubMed

Table of findings - Eating disorders

Study	Objective/methods	Main results	Conclusions
Jacobi C, Völker U, Trockel MT, Taylor CB. Effects of an Internet-based intervention for subthreshold eating disorders: a randomized controlled trial. Behav Res Ther. 2012;50(2):93-9.	To adapt and evaluate the effects of the Internet-based prevention program Student Bodies™ for women with symptoms of disordered eating and/or subthreshold ED syndromes. 126 women, reporting subthreshold ED symptoms (high weight and shape concerns and below threshold bingeing, purging, chronic dieting or several of these symptoms) were randomly assigned to a Student Bodies™+ (SB+) intervention or a wait-list control group and assessed at pre-intervention, post-intervention, and 6-month follow-up. Student Bodies™ was adapted to be suitable for subthreshold ED.	At 6-month follow-up, compared to participants in the control group, participants in the intervention group showed significantly greater improvements on ED-related attitudes. Intervention participants also showed 67% (95%CI=20-87%) greater reductions in combined rates of subjective and objective binges, and 86% (95%CI=63-95%) greater reduction in purging episodes. Also, the rates of participants abstinent from all symptoms of ED (restrictive eating, binge eating and any compensatory behavior) were significantly higher in the intervention group (45.1% *versus* 26.9%). Post-hoc subgroup analyses revealed that for participants with binge eating the effect on EDE-Q scores was larger than in the pure restricting subgroup.	The adapted SB+ program represents an effective intervention for women with subthreshold ED of the binge eating subtype.
Geller J, Brown KE, Srikameswaran S. The efficacy of a brief motivational intervention for individuals with eating disorders: a randomized control trial. Int J Eat Disord. 2011;44(6):497-505.	To determine the efficacy of (RMT), a five-session individual preparatory intervention for individuals with ED. Participants completed the readiness and motivation interview and measures of ED symptomatology, self-esteem, and psychiatric symptoms at intake. A total of 181 participants were randomly assigned to the treatment (RMT) or wait-list control condition and were reassessed at 6-week and 3-month follow-up; 113 completed assessments at all three time points and primary analyses were based on these individuals.	Surprisingly, improvements in readiness for change, depression, drive for thinness, and bulimia symptoms occurred over time in both RMT and control conditions. However, at post-treatment and at follow-up, individuals who received RMT were less likely to have high ambivalence than were those from the control condition.	RMT may be of benefit to highly reluctant, clinically challenging patients and help them make better use of future, action-oriented treatment.
Vocks S, Schulte D, Busch M, Grönemeyer D, Herpertz S, Suchan B. Changes in neuronal correlates of body image processing by means of cognitive-behavioural body image therapy for eating disorders: a randomized controlled fMRI study. Psychol Med. 2011;41(8):1651-63.	To analyze treatment-induced changes in neuronal correlates of visual body image processing. Thirty-two females with ED were randomly assigned either to a manualized CBT body image consisting of ten group sessions, or to a waiting list control condition. Using functional magnetic resonance imaging, brain responses to viewing photographs of one's own and another female's body taken from 16 standardized perspectives while participants were wearing a uniform bikini were acquired before and after the intervention and the waiting time, respectively.	Data indicate a general blood oxygen level dependent signal enhancement in response to looking at photographs of one's own body from pre- to post-treatment, whereas exclusively in the control group activation decreases from pre- to post-waiting time were observed. Focused activation increases from pre- to post-treatment were found in the left middle temporal gyrus covering the coordinates of the extrastriate body area and in bilateral frontal structures including the middle frontal gyrus.	Results point to a more intense neuronal processing of one's own body after the CBT body image in cortical regions that are responsible for the visual processing of the human body and for self-awareness.
Catalan-Matamoros D, Helvik-Skjaerven L, Labajos-Manzanares MT, Martínez-de-Salazar-Arboleas A, Sánchez-Guerrero E. A pilot study on the effect of Basic Body Awareness Therapy in patients with eating disorders: a randomized controlled trial. Clin Rehabil. 2011;25(7):617-26.	To analyse the feasibility of basic body awareness therapy in people ED. Evaluation of 28 outpatients with ED for less than 5 years. All patients received standard outpatient treatment. The intervention group (n=14) also received basic body awareness therapy for 7 weeks.	Analysing the differences between both groups, significant differences were found in Eating Disorder Inventory (mean difference: 26.3; p=0.015) and in its subscales 'drive to thinness' (p=0.003), 'body dissatisfaction' (p=0.025) and 'ineffectiveness' (p=0.014). Also in Body Attitude Test (mean difference: 33.0; p=0.012), Eating Attitude Test-40 (mean difference: 17.7; p=0.039) and SF-36 in the section 'mental health' (mean difference: 13.0; p=0.002).	This study showed some effectiveness of basic body awareness therapy in improving some symptoms in outpatients with ED. Further studies should include larger samples, double-blinded and placebo methodologies, and should focus on questions such as which ED diagnoses benefit most from physical therapy.

continue...

...Continuation

Extracted of PubMed

Table of findings - Eating disorders

Study	Objective/methods	Main results	Conclusions
Byrne SM, Fursland A, Allen KL, Watson H. The effectiveness of enhanced cognitive behavioural therapy for eating disorders: an open trial. Behav Res Ther. 2011;49(4):219-26.	To examine the effectiveness of CBT-E for ED in an open trial for adults with the full range of ED found in the community. The current study represents the first published trial of CBT-E to include patients with a BMI<17.5. The study involved 125 patients referred to a public outpatient clinic in Perth, Western Australia. Patients attended, on average, 20 to 40 individual sessions with a clinical psychologist.	Of those who entered the trial, 53% completed treatment. Longer waiting time for treatment was significantly associated with dropout. By the end of treatment, full remission (cessation of all key ED behaviours, BMI≥18.5kg/m^2, not meeting DSM-IV criteria for an ED) or partial remission (meeting at least two of these criteria) was achieved by two thirds of patients who completed.treatment and 40% of the total sample. The results compared favourably to those reported in the previous RCT of CBT-E, with one exception being the higher dropout rate in the current study.	Overall, the findings indicated that CBT-E results in significant improvements, in both eating and more general psychopathology, in patients with all ED attending an outpatient clinic.
Smeets E, Tiggemann M, Kemps E, Mills JS, Hollitt S, Roefs A, et al. Body checking induces an attentional bias for body-related cues. Int J Eat Disord. 2011;44(1):50-7.	To examine the influence of body checking on attentional bias for body-related cues by manipulating body checking behaviors in nonclinical participants. Randomization of 66 women to one of three conditions: body checking, body exposure, or control. A body visual search task was used to measure attentional bias.	Participants in the body checking condition showed speeded detection of body-related information compared to participants in the exposure and control conditions. No evidence was found for increased distraction by body-related information. Furthermore, participants in the body checking condition reported more body dissatisfaction after the manipulation than participants in the body exposure and control conditions.	These results are the first to experimentally establish the link between body checking and attentional bias toward body-related cues.
Traviss GD, Heywood-Everett S, Hill AJ. Guided self-help for disordered eating: A randomised control trial. Behav Res Ther. 2011;49(1):25-31.	The current randomised control trial evaluated a cognitive behavioural therapy-based GSH pack, Working to Overcome Eating Difficulties, delivered by trained mental health professionals in 6 sessions over 3 months. It was congruent with the transdiagnostic approach and so was intended as suitable for all disordered eating, except severe AN. Eighty one clients were randomly allocated to either a GSH or waiting list condition. ED psychopathology (EDE-Q), key behavioural features and global distress (CORE) were measured at pre- and post-intervention, and 3- and 6-month follow-up.	Results showed significant improvements in ED psychopathology, laxative abuse, exercise behaviours, and global distress, with the GSH condition being superior to the waiting list on all outcomes. Treatment gains were maintained at 3 and 6 months.	This study adds to the evidence supporting GSH for disordered eating, including EDNOS. However, further work is needed to establish the factors that contribute to observed therapeutic improvements and to determine for whom GSH is most suitable.
Storch M, Keller F, Weber J, Spindler A, Milos G. Psychoeducation in affect regulation for patients with eating disorders: a randomized controlled feasibility study. Am J Psychother. 2011;65(1):81-93.	To examine the effects of a psychoeducational training program in affect regulation for patients with ED. A total of 19 female patients completed measures of affect regulation (ACS-90), alexithymia (TAS-26), and eating behavior (EDE-Q). Data were assessed at baseline and at 3 and 12 months after treatment. Dependent on date of entering hospital, the patients were allocated consecutively to the control group (n=11), which received inpatient treatment as usual, or to the treatment group (n=8), which received training in addition to usual inpatient treatment.	At follow-up, the training was associated with statistically significant improvement in the skill of down-regulating negative affect and with a tendency towards less dietary restraint. Regarding alexithymia no clear results were found.	Despite the small sample size, results showed that in addition to the general improvement due to the treatment usually provided in the clinical setting, the training program resulted in specific benefits for the patients with regard to their affect regulation skills.
Dunker KL, Philippi ST, Ikeda JP. Interactive Brazilian program to prevent eating disorders behaviors: a pilot study. Eat Weight Disord. 2010;15(4):e270-4.	During a four month scholarly leave in United States, researchers designed a culturally appropriate prevention program for ED for Brazilian adolescent girls. The program *Se Liga na Nutrição* was modeled on other effective programs identified in a research literature review and was carried out over eleven interactive sessions.	It was positively received by the adolescents who suggested that it be part of school curricula. The girls reported that it helped them to develop critical thinking skills with regards to sociocultural norms about body image, food and eating practices.	

continue...

...Continuation

Extracted of PubMed

Table of findings - Eating disorders

Study	Objective/methods	Main results	Conclusions
Becker CB, Wilson C, Williams A, Kelly M, McDaniel L, Elmquist J. Peer-facilitated cognitive dissonance versus healthy weight eating disorders prevention: A randomized comparison. Body Image. 2010;7(4):280-8.	Research supports the efficacy of both (CD) and healthy weight ED, and indicates that CD can be delivered by peer-facilitators, which facilitates dissemination.This study investigated if peer-facilitators can deliver healthy weight when it is modified for their use and extended follow-up of peer-facilitated CD as compared to previous trials. Based on pilot data, we modified healthy weight to facilitate peer delivery, elaborate benefits of the healthy-ideal, and place greater emphasis on consuming nutrient dense foods. Female sorority members (n=106) were randomized to either two 2-hour sessions of CD or modified healthy weight. Participants completed assessment pre- and post-intervention, and at 8-week, 8-month, and 14-month follow-up.	Consistent with the hypotheses, CD decreased negative affect, thin-ideal internalization, and bulimic pathology to a greater degree post-intervention. Both CD and modified healthy weight reduced negative affect, internalization, body dissatisfaction, dietary restraint, and bulimic pathology at 14 months.	
East P, Startup H, Roberts C, Schmidt U. Expressive writing and eating disorder features: a preliminary trial in a student sample of the impact of three writing tasks on eating disorder symptoms and associated cognitive, affective and interpersonal factors. Eur Eat Disord Rev. 2010;18(3):180-96.	To evaluate the impact of three writing tasks on the cognitive, affective and interpersonal factors typically associated with ED symptoms, in a student population. Two experimental tasks and one control task were evaluated.	Participants gave subjective ratings of the writing experience, and objective questionnaire measures were administered at baseline, and 4- and 8-week follow-up. Participants who dropped out without completing the writing tasks were more experientially avoidant. The three tasks differed significantly in subjective impact, and the experimental tasks were more effective in reducing ED symptoms. They also ameliorated some key features associated with eating difficulties. The control task generally had less, no or a detrimental effect.	The results provide preliminary indirect support for the use of therapeutic writing to address specific features associated with the ED presentation. Further research is required to replicate the present findings and extend these to the clinical population.
Juarascio AS, Forman EM, Herbert JD. Acceptance and commitment therapy versus cognitive therapy for the treatment of comorbid eating pathology. Behav Modif. 2010;34(2):175-90.	To examine several questions related to the treatment of eating pathology within the context of a larger RCT that compared standard CBT (*i.e.*, Beck's cognitive therapy; CT) with acceptance and commitment therapy (Hayes, 2004).	The results indicated that the two treatments were differentially effective at reducing eating pathology. Specifically, CT produced modest decreases in eating pathology whereas acceptance and commitment therapy produced large decreases. In addition, a weaker suggestion emerged that acceptance and commitment therapy was more effective than CT at increasing clinician-rated global functioning among those with eating pathology.	These findings suggest that acceptance and commitment therapy is a useful treatment for disordered eating and potentially, for ED *per se*.
Allison KC, Lundgren JD, Moore RH, O'Reardon JP, Stunkard AJ. Cognitive behavior therapy for night eating syndrome: a pilot study. Am J Psychother. 2010;64(1):91-106.	To conduct a pilot study of a 10-session CBT for NES. A total of 25 patients (19 female) were screened and comprehensively assessed before being enrolled. At each visit, patients completed the NESS, were weighed, and number of awakenings and the number of nocturnal ingestions and daily caloric intake were calculated from weekly food and sleep records.	Mixed model regression analyses of the data showed significant decreases in caloric intake after dinner (35.0% to 24.9%); number of nocturnal ingestions (8.7 to 2.6 per week); weight (82.5 to 79.4kg); and NESS score (28.7 to 16.3; all p values <0.0001). Number of awakenings per week, depressed mood, and quality of life also improved significantly (p values <0.02).	This first clinical trial of CBT for NES shows significant improvements in the core aspects of NES and weight reduction, suggesting the need for a controlled treatment trial.

continue...

...Continuation

Extracted of PubMed

Table of findings - Eating disorders

Study	Objective/methods	Main results	Conclusions
Provencher V, Bégin C, Tremblay A, Mongeau L, Corneau L, Dodin S, et al. Health-At-Every-Size and eating behaviors: 1-year follow-up results of a size acceptance intervention. J Am Diet Assoc. 2009;109(11):1854-61.	To assess the effects of HAES intervention on eating behaviors, appetite sensations, metabolic and anthropometric variables, and physical activity levels in women at 6-month and 1-year after intervention. Premenopausal overweight/obese women (n=144; mean age of 42.3±5.6 years), recruited from free-living, general community women were randomly assigned to: HAES group (n=48), social support group (n=48), or control group (n=48).	Situational susceptibility to disinhibition and susceptibility to hunger significantly decreased over time in both HAES group (-0.9±0.2 and -1.3±0.5, respectively) and the social support group (-0.4±0.2 and -1.4±0.5, respectively). Although eating behavior scores observed at 16 months did not differ between HAES and social support groups (situational susceptibility to disinhibition: 2.5±0.2 in HAES group *versus* 2.7±0.2 in social support group; susceptibility to hunger: 4.2±0.5 in both groups), they were lower in these groups than scores noted in the control group (3.3±0.2 for situational susceptibility to disinhibition and 5.9±0.5 for susceptibility to hunger).	These results suggest that, when compared to a control group, an HAES approach could have long-term beneficial effects on eating behaviors related to disinhibition and hunger. In addition, our study did not show distinctive effects of the HAES approach in comparison to a social support intervention.
Korrelboom K, de Jong M, Huijbrechts I, Daansen P. Competitive memory training (COMET) for treating low self-esteem in patients with eating disorders: A randomized clinical trial. J Consult Clin Psychol. 2009;77(5):974-80.	This study evaluates a short stepwise cognitive-behavioral intervention for the treatment of low self-esteem in patients with ED. Competitive memory training for low self-esteem is based on insights and findings from experimental psychology. A total of 52 patients with ED and low self-esteem were treated with competitive memory training in a routine mental health center in addition to their regular treatment. These patients were randomized to receive 8 weeks of competitive memory training + therapy as usual or to receive therapy as usual only.	Effects in favor of competitive memory training + therapy as usual were found for two indexes of self-esteem and for one index of depressive mood.	Shortcomings of this study and possible clinical implications are discussed.
Wilksch SM, Wade TD. Reduction of shape and weight concern in young adolescents: a 30-month controlled evaluation of a media literacy program. J Am Acad Child Adolesc Psychiatry. 2009;48(6):652-61.	To evaluate a theoretically informed media literacy program delivered to a mixed-sex, universal, young adolescent audience. Five hundred forty Grade 8 students (mean age 13.62 years, SD of 0.37 years) from 4 schools participated with a total of 11 classes receiving the 8-lesson media literacy program (126 girls and 107 boys) and 13 comparison classes receiving their normal school. lessons (147 girls and 160 boys). Shape and weight concern (primary outcome variable) and seven additional ED risk factors (*e.g.*, dieting, media internalization) were measured with validated questionnaires at baseline, postprogram, and 6- and 30-month follow-up.	Linear mixed model analyses were conducted using a 2 (group: media literacy program, control) x 3 (time: postprogram, 6-month follow-up, 30-month follow-up) x 2 (sex: girls, boys) mixed within-between design, with baseline entered as a covariate. Main effects for group, favoring the media literacy program, were found for shape and weight concern (effect size of 0.29), dieting (effect size of 0.26), body dissatisfaction (effect size of 0 0.20), ineffectiveness (effect size of 0 0.23), and depression (effect size of 0 0.26).	Media literacy can be an effective intervention for reducing shape and weight concern and other ED risk factors long-term in a universal mixed-sex, young adolescent population. More evaluations of methodologically sound prevention programs are required with this demographic.
Fairburn CG, Cooper Z, Doll HA, O'Connor ME, Bohn K, Hawker DM, et al. Transdiagnostic cognitive-behavioral therapy for patients with eating disorders: a two-site trial with 60-week follow-up. Am J Psychiatry. 2009;166(3):311-9.	To compare two CBT for outpatients with ED, one focusing solely on ED features and the other a more complex treatment that also addresses mood intolerance, clinical perfectionism, low self-esteem, or interpersonal difficulties.patients who had a DSM-IV ED but were not markedly underweight (BMI over 17.5), were enrolled in a two-site RCT involving 20 weeks of treatment and a 60-week closed period of follow-up. The control condition was an 8-week waiting list period preceding treatment.	Patients in the waiting list control condition exhibited little change in symptom severity, whereas those in the two treatment conditions exhibited substantial and equivalent change, which was well maintained during follow-up. At the 60-week follow-up assessment, 51.3% of the sample had a level of ED features less than one standard deviation above the community mean. Treatment outcome did not depend on ED diagnosis. Patients with marked mood intolerance, clinical perfectionism, low self-esteem, or interpersonal difficulties appeared to respond better to the more complex treatment, with the reverse pattern evident among the remaining patients.	

continue...

...Continuation

Extracted of PubMed

Table of findings - Eating disorders

Study	Objective/methods	Main results	Conclusions
Doyle AC, Goldschmidt A, Huang C, Winzelberg AJ, Taylor CB, Wilfley DE. Reduction of overweight and eating disorder symptoms via the Internet in adolescents: a randomized controlled trial. J Adolesc Health. 2008;43(2):172-9.	This multisite RCT evaluated an Internet-delivered program targeting weight loss and ED attitudes/behaviors in adolescents. A total of 80 overweight adolescents 12-17 years of age completed Student Bodies 2, a 16-week cognitive-behavioral program, or usual care.	BMI Z scores were reduced in the Student Bodies 2 group compared with the usual care group from baseline to post-intervention (p=0.027; eta(p)(2)=0.08). The Student Bodies 2 group maintained this reduction in BMI Z scores at 4-month follow-up, but significant differences were not observed because of improvement in the usual care group. The Student Bodies 2 group evidenced greater increases in dietary restraint post-intervention (p ==0.016) and less improvement on shape concerns at follow-up (p=0.044); however these differences were not clinically significant. No other statistically significant differences were noted between groups on ED attitudes or behaviors. The Student Bodies 2 participants reported using healthy eating-related and physical activity-related skills more frequently than usual care participants post-intervention (p=0.001) and follow-up (p=0.012).	Findings suggest that an Internet-delivered intervention yielded a modest reduction in weight status that continued 4 months after treatment and that ED attitudes/behaviors were not significantly improved. Group differences on weight loss were not sustained at 4-month follow-up because of parallel improvements in the groups. Future studies are needed to improve program adherence and to further explore the efficacy of Internet-delivery of weight control programs for adolescents.
Dohnt HK, Tiggemann M. Promoting positive body image in young girls: an evaluation of 'Shapesville'. Eur Eat Disord Rev. 2008;16(3):222-33.	To evaluate Shapesville, a children's picture book designed to promote positive body image in young children. Participants were a convenience sample comprising 84 girls (aged 5-9 years) recruited from four private girls' schools. Girls were randomly allocated to be read either Shapesville or a control book. Individual measures of body image, stereotyping on the basis of weight and media internalisation, as well as knowledge of non-appearance topics, were obtained at pre- and post-intervention, as well as at 6-week follow-up.	Relative to the control book, girls' appearance satisfaction increased after reading Shapesville. In addition, stereotyping on the basis of weight and internalisation of media ideals was reduced. Finally, reading Shapesville also increased girls' knowledge base of non-appearance topics, such as recognising their special talents and awareness of healthy eating, at post-intervention. These gains were somewhat reduced at follow-up, yet were largely still significantly greater than at pre-intervention.	The present study demonstrates that Shapesville has the potential to be a successful prevention tool for use with young girls. Schools can incorporate Shapesville into their curriculum as early as school entry, in order to help prevent the early development of body dissatisfaction and disordered eating.
Hay P, Mond J, Paxton S, Rodgers B, Darby A, Owen C. What are the effects of providing evidence-based information on eating disorders and their treatments? A randomized controlled trial in a symptomatic community sample. Early Interv Psychiatry. 2007;1(4):316-24.	We hypothesize that a reason for the infrequent uptake of treatments by people with ED is poor knowledge about treatments and outcomes for ED (ED - Mental Health Literacy – ED-MHL). Our aim was to test putative health benefits of a brief ED-MHL intervention. In a community-based two-phase survey, 122 young women (mean age 28.5, SD 6.3 years) with ED symptoms meeting DSM-IV criteria for clinical severity were randomized to receive either a brief ED-MHL intervention (comprising information about efficacious treatments, reputable self-help books and where to go for further information and/or services) or information about local mental health services only. All were given feedback on their scores on measures of ED symptoms and quality of life.	102 participants (84%) completed follow-up at 12 months. Symptomatic improvement and changes in specific aspects of ED-MHL, namely, less pessimism about how difficult ED are to treat and improved recognition and knowledge, as well as increased help seeking, were observed in both groups. Differences between groups were uncommon but compared with control participants, those in the intervention group had improved health-related quality of life.	A brief community-based intervention aimed to improve knowledge and beliefs about ED and their treatments may be a valuable first step in improving health-related outcomes for people with ED, but more research is needed.

continue...

...Continuation

Extracted of PubMed

Table of findings - Eating disorders

Study	Objective/methods	Main results	Conclusions
Heinicke BE, Paxton SJ, McLean SA, Wertheim EH. Internet-delivered targeted group intervention for body dissatisfaction and disordered eating in adolescent girls: a randomized controlled trial. J Abnorm Child Psychol. 2007;35(3):379-91.	This study evaluated a targeted intervention designed to alleviate body image and eating problems in adolescent girls that was delivered over the internet so as to increase access to the program. The program consisted of six, 90-minute weekly small group, synchronous on-line sessions and was facilitated by a therapist and manual. Participants were 73 girls (mean age=14.4 years, SD=1.48) who self-identified as having body image or eating problems and were randomly assigned to an intervention group (n=36) (assessed at baseline, post-intervention and at 2- and 6-month follow-up) or a delayed treatment control group (n=37) (assessed at baseline and 6-7 weeks later).		The program offers a promising approach to improve body image and eating problems that also addresses geographic access problems.
Mitchell KS, Mazzeo SE, Rausch SM, Cooke KL. Innovative interventions for disordered eating: evaluating dissonance-based and yoga interventions. Int J Eat Disord. 2007;40(2):120-8.	Eating-disordered behavior is prevalent among college women. Few interventions have successfully reduced risk factors for these behaviors, however. The most promising interventions are both selective and interactive. This study compared two newer types of interventions that meet these criteria: cognitive dissonance and yoga programs. This study advertised programs for women who were dissatisfied with their bodies. Participants (N = 93) were randomly assigned to dissonance, yoga, or control groups.	Hierarchical regression analyses revealed that there were no significant post-intervention differences between the yoga and control groups. Dissonance group participants had significantly lower scores than the scores of both other groups on measures of disordered eating, drive for thinness, body dissatisfaction, alexithymia, and anxiety.	These findings have important implications for interventions on college campuses. In particular, dissonance interventions appear to be an efficient and inexpensive approach to reducing ED risk factors. Additional research regarding the value of yoga interventions is needed.
Jacobi C, Morris L, Beckers C, Bronisch-Holtze J, Winter J, Winzelberg AJ, et al. Maintenance of internet-based prevention: a randomized controlled trial. Int J Eat Disord. 2007;40(2):114-9.	To determine the short-term and maintenance effects of an internet-based prevention program for ED. One hundred female students at two German universities were randomly assigned to either an 8-week intervention or a waiting-list control condition and assessed at pre-intervention, post-intervention, and 3-month follow-up.	Compared with the control group, the intervention produced significant and sustained effects for high-risk women.	Internet-based prevention is effective and can be successfully adapted to a different culture.
Richards PS, Berrett ME, Hardman RK, Eggett DL. Comparative efficacy of spirituality, cognitive, and emotional support groups for treating eating disorder inpatients. Eat Disord. 2006;14(5):401-15.	To evaluate the effectiveness of a spiritual group intervention for ED inpatients. We compared the effectiveness of a spirituality group with cognitive and emotional support groups using a randomized, control group design. Participants were 122 women receiving inpatient ED treatment.	Patients in the spirituality group tended to score significantly lower on psychological disturbance and ED symptoms at the conclusion of treatment compared to patients in the other groups, and higher on spiritual well-being. On weekly outcome measures, patients in the Spirituality group improved significantly more quickly during the first 4 weeks of treatment.	This study provides preliminary evidence that attending to ED patients' spiritual growth and well-being during inpatient treatment may help reduce depression and anxiety, relationship distress, social role conflict, and ED symptoms.
Stice E, Shaw H, Burton E, Wade E. Dissonance and healthy weight eating disorder prevention programs: a randomized efficacy trial. J Consult Clin Psychol. 2006;74(2):263-75.	Adolescent girls with body dissatisfaction (n=481, mean age =17 years) were randomized to an ED prevention program involving dissonance-inducing activities that reduce thin-ideal internalization, a prevention program promoting healthy weight management, an expressive writing control condition, or an assessment-only control condition.	Dissonance participants showed significantly greater reductions in ED risk factors and bulimic symptoms than healthy weight, expressive writing, and assessment-only participants, and healthy weight participants showed significantly greater reductions in risk factors and symptoms than expressive writing and assessment-only participants from pre-test to post-test.	Although these effects faded over 6-month and 12-month follow-ups, dissonance and healthy weight participants showed significantly lower binge eating and obesity onset and reduced service utilization through 12-month follow-up, suggesting that both interventions have public health potential.

continue...

...Continuation

Extracted of PubMed

Table of findings - Eating disorders

Study	Objective/methods	Main results	Conclusions
Gollings EK, Paxton SJ. Comparison of internet and face-to-face delivery of a group body image and disordered eating intervention for women: a pilot study. Eat Disord. 2006;14(1):1-15.	Increased access to therapy for body dissatisfaction and disordered eating is required. This pilot study compared a group intervention delivered face-to-face or synchronously over the Internet. Women with body dissatisfaction and disordered eating were randomly assigned to a face-to-face (n=19) or Internet (n=21) group.	Body dissatisfaction, disordered eating, and psychological variables were assessed at baseline, post-intervention, and 2 months follow-up. Significant improvements on all outcome variables were observed and maintained at follow-up in both groups. There were no significant differences between delivery modes.	This program shows promise, and the Internet mode of delivery has potential to overcome geographical distance.
Kong S. Day treatment programme for patients with eating disorders: randomized controlled trial. J Adv Nurs. 2005;51(1):5-14.	To compare the effects of day treatment programmes for patients with ED with those of traditional outpatient treatment. Volunteers from an outpatient clinic for ED were randomly assigned either to a treatment group (n=21), participating in a modified day treatment programme based on the Toronto Day Hospital Program, or to a control group (n=22) receiving a traditional. outpatient programme of IPT, cognitive behaviour therapy and pharmacotherapy.	Participants in the day treatment programme showed significantly greater improvements on most psychological symptoms of the Eating Disorder Inventory-2, frequency of binging and purging, body mass index, depression and self-esteem scores than the control group. They also showed significant improvement in perfectionism, but the group difference was not significant.	Nurses in day treatment programmes can play various and important roles establishing a therapeutic alliance between patient and carer in the initial period of treatment. In addition, the cognitive and behavioural work that is vital to a patient's recovery, that is, dealing with food issues, weight issues and self-esteem, is most effectively provided by a nurse therapist who maintains an empathic involvement with the patient.
Matusek JA, Wendt SJ, Wiseman CV. Dissonance thin-ideal and didactic healthy behavior eating disorder prevention programs: results from a controlled trial. Int J Eat Disord. 2004;36(4):376-88.	In the current study, college women with body image concerns (n=84) were randomly assigned to a cognitive dissonance-based, thin-ideal internalization, single-session workshop (n=26); a psychoeducational, healthy behavior, single-session workshop (n=24); or a wait-list control (n=34).	Comparing baseline data with 4-week follow-up data, results indicated that both cognitive dissonance-based, thin-ideal internalization and healthy behavior participants reported improvement in body image, thin-ideal internalization, and eating behaviors.	Results provide evidence that both interventions - cognitive dissonance-based, thin-ideal internalization, single-session workshop (n=26) and a psychoeducational, healthy behavior, single-session workshop (n=24) - effectively reduce risk factors for eating pathology.
Pawlow LA, O'Neil PM, Malcolm RJ. Night eating syndrome: effects of brief relaxation training on stress, mood, hunger, and eating patterns. Int J Obes Relat Metab Disord. 2003;27(8):970-8.	To determine whether a Relaxation Intervention (abbreviated progressive muscle relaxation therapy) that has been shown to significantly reduce stress levels in normal, healthy adults would also benefit an NES sample. A total of 20 adults with NES were randomly assigned to either a relaxation training or a control (quietly sitting for the same amount of time) group, and all subjects attended two laboratory sessions, one week apart. Pre- and post-session indices of stress, anxiety, relaxation, and salivary cortisol were obtained, as well as Day 1 and Day 8 indices of mood. Food diaries and hunger ratings were also obtained.	The results indicated that 20 minutes of a muscle relaxation exercise significantly reduced stress, anxiety, and salivary cortisol immediately postsession. After practicing these exercises daily for a week, subjects exhibited lowered stress, anxiety, fatigue, anger, and depression on day 8. Abbreviated progressive muscle relaxation therapy was also associated with significantly higher a.m. and lower p.m. ratings of hunger, and a trend of both more breakfast and less night-time eating.	These data support the role of stress and anxiety in NES and suggest that practicing relaxation may be an important component of treatment for this condition.
**Systematic review/meta-analysis**	**Objective/methods**	**Main results**	**Conclusions**
Spielmans GI, Benish SG, Marin C, Bowman WM, Menster M, Wheeler AJ. Specificity of psychological treatments for bulimia nervosa and binge eating disorder? A meta-analysis of direct comparisons. Clin Psychol Rev. 2013;33(3):460-9. Review.	Treatment guidelines state that CBT and interpersonal therapy are the best-supported psychotherapies for BN and that CBT is the preferred psychological treatment for BED. However, no meta-analysis with both examined direct comparisons between psychological treatments for BN and BED and considered the role of moderating variables, such as the degree to which psychotherapy was bona fide, has previously been conducted. Thus, such an analysis was undertaken. We included 77 comparisons reported in 53 studies.	The results indicated that: (a) bona fide therapies outperformed non-bona fide treatments, (b) bona fide CBT outperformed bona fide non-CBT interventions by a statistically significant margin (only approaching statistical significance for BN and BED when examined individually), but many of these trials had confounds which limited their internal validity, (c) full CBT treatments offered no benefit over their components, and (d) the distribution of effect size differences between bona fide CBT treatments was homogeneously distributed around zero.	These findings provide little support for treatment specificity in psychotherapy for BN and BED.

continue...

...Continuation

Extracted of PubMed

Table of findings - Eating disorders

Systematic review/meta-analysis	Objective/methods	Main results	Conclusions
Berner LA, Allison KC. Behavioral management of night eating disorders. Psychol Res Behav Manag. 2013;6:1-8. Review.	NES is a form of disordered eating associated with evening hyperphagia (overeating at night) and nocturnal ingestions (waking at night to eat). As with other forms of disordered eating, cognitive and behavioral treatment modalities may be effective in reducing NES symptoms.	This review presents evidence for a variety of behavioral treatment approaches, including behavioral therapy, phototherapy, behavioral weight loss treatment, and CBT. A more detailed overview of CBT for NES is provided. All of these studies have been case studies or included small samples, and all but one have been uncontrolled. Nonetheless, the outcomes of many of these approaches are promising.	Larger RCT are warranted to advance NES treatment literature. With the inclusion of NES in the fifth edition of the DSM-5 as a "feeding or ED not elsewhere classified," more sophisticated, empirically-supported, behaviorally-based treatment approaches are much needed.
Hausenblas HA, Campbell A, Menzel JE, Doughty J, Levine M, Thompson JK. Media effects of experimental presentation of the ideal physique on eating disorder symptoms: a meta-analysis of laboratory studies. Clin Psychol Rev. 2013;33(1):168-81. Review.	These meta-analyses included correlational, quasi-experimental, and experimental studies, with limited examination of moderators and other relevant outcomes besides body image. A total of 33 experimental studies were identified (*i.e.*, pre- and post-data for both experimental and control groups). The laboratory studies examined the effects of acute exposure to the media's portrayal of the ideal physique on ED symptoms (*i.e.*, body image, positive affect, negative affect, self-esteem, anger, anxiety and depression) and the mechanisms that moderate this effect.	Fourteen separate meta-analyses revealed a range of small to moderate effect sizes for change in outcomes from pre- to post-treatment for both experimental and control groups. Exposure to images of the ideal physique resulted in small effect sizes for increased depression and anger and decreased self-esteem and positive affect. Moderator analyses revealed moderate effect sizes for increased depression and body dissatisfaction among high-risk participants.	This meta-analysis makes it clear that media exposure of the ideal physique results in small changes in ED symptoms, particularly with participants at high risk for developing an ED. Further research is needed to examine the longitudinal effects of media exposure of ED symptoms.
Couturier J, Kimber M, Szatmari P. Efficacy of family-based treatment for adolescents with eating disorders: a systematic review and meta-analysis. Int J Eat Disord. 2013;46(1):3-11. Review.	The study aims to systematically review the literature as it pertains to family therapies that follow Maudsley principles for adolescents with ED, and to compile the results quantitatively using meta-analysis. Twelve RCT were included involving adolescents with ED and family therapy which were reviewed carefully for several inclusion criteria including: allocation concealment, intent-to-treat analysis, assessor blinding, behavioral family therapy compared with an individual therapy, and adolescent age group. References from these articles were searched. Only three studies met these strict inclusion criteria for meta-analysis. A random effects model and odds ratio was used for meta-analysis, looking at "remission" as the outcome of choice.	When combined in a meta-analysis, end of treatment data indicated that FBT was not significantly different from individual treatment ($Z=1.62$; $p=0.11$). When follow-up data from 6 to 12 months were analyzed, FBT was superior to individual treatment ($Z=2.94$, $p<0.003$), and heterogeneity was not significant ($p=0.59$).	Although FBT does not appear to be superior to individual treatment at end of treatment, there appear to be significant benefits at 6 to 12 month follow-up for adolescents suffering from ED.
Hay PJ, Claudino AM. Clinical psychopharmacology of eating disorders: a research update. Int J Neuropsychopharmacol. 2012;15(2):209-22. Review.	The paper presents a critical review (with search date 2010) of the major psychotropic medications assessed in ED, namely antipsychotics, antidepressants, mood-stabilizing medications, anxiolytic and other agents.	The evidence of efficacy of drug treatments is mostly weak or moderate. In addition, attrition rates are usually higher than for psychotherapies. However, there is support for use of antidepressants, particularly high-dose fluoxetine in BN, and anticonvulsants (topiramate) for BED. Low-dose antipsychotic medication may be clinically useful as adjunct treatment in acute anorexia, particularly where there is high anxiety and obsessive eating-related ruminations and failure to engage, but more trials are needed. Drug therapies, such as topiramate and anti-obesity medication, may aid weight loss in obese or overweight patients with binge-ED; however, common or potentially serious adverse effects limit their use.	

continue...

...Continuation

Extracted of PubMed

Table of findings - Eating disorders

Systematic review/meta-analysis	Objective/methods	Main results	Conclusions
Allen S, Dalton WT. Treatment of eating disorders in primary care: a systematic review. J Health Psychol. 2011;16(8):1165-76. Review.	This review evaluated psychological treatments for AN, BN, and BED conducted in primary care. Five studies met inclusion criteria.	CBT-GSH book may be a beneficial, first-line treatment for reducing binging and purging symptoms. Outcomes combining self-help with antidepressants remain unclear, although antidepressants alone may provide reduction of symptoms. High attrition and non-compliance rates among studies reviewed indicate the importance of a strong therapeutic alliance between provider and patient.	Further research in primary care is needed to develop a standard of care for patients with ED.
Herpertz S, Hagenah U, Vocks S, von Wietersheim J, Cuntz U, Zeeck A; German Society of Psychosomatic Medicine and Psychotherapy; German College for Psychosomatic Medicine. The diagnosis and treatment of eating disorders. Dtsch Arztebl Int. 2011;108(40):678-85.	This scientifically based S3 guideline was developed with the intention of improving the treatment of ED and motivating future research in this area. The existing national and international guidelines on the three types of ED were synoptically compared, the literature on the subject was systematically searched, and meta-analyses on BN and CBT-E were carried out. Fifteen consensus conferences were held, and the results were 44 evidence-based recommendations issued. Anorexia and BN are diagnosed according to the ICD-10 criteria, CBT-E according to those of the DSM.	Psychotherapy is the mainstay of treatment for all three disorders, and Cognitive Behavioral Therapy is the form of psychotherapy best supported by the available evidence. The administration of SSRI can be recommended as a flanking measure in the treatment of BN only. The evidence does not support any type of pharmacotherapy for AN or binge-ED. BN and BED can usually be treated on an outpatient basis, as long as they are no more than moderately severe; full-fledged AN is generally an indication for in-hospital treatment.	This guideline contains evidence- and consensus-based recommendations for the diagnosis and treatment of ED. If strictly implemented, it should result in improved care for the affected patients.
Hart LM, Granillo MT, Jorm AF, Paxton SJ. Unmet need for treatment in the eating disorders: A systematic review of eating disorder specific treatment seeking among community cases. Clin Psychol Rev. 2011;31(5):727-35. Review.	To systematically review the literature on the proportion of community cases with a diagnosable ED who seek ED specific treatment. A total of 14 articles met inclusion criteria, comprising 1581 participants with a diagnosable ED.	The pooled proportion seeking treatment was 23.2% (95%CI=16.6-31.4), however this estimate was associated with significant variability. The proportion seeking treatment for weight loss ranged from 30 to 73%, indicating that individuals with ED are much more likely to receive treatment for a perceived problem with weight.	The literature provides a complex picture, as a minority receive appropriate mental health care, yet many receive treatment for weight loss. Significant gaps in the literature currently exist and future research needs to focus on treatment seeking in the young and elderly, males, and in countries outside of Australia and the United States. There is a need for interventions that assist community members, health professionals and treatment services to recognize ED and understand their associated burden and the benefit of providing appropriate and timely treatment.
Wilfley DE, Kolko RP, Kass AE. Cognitive-behavioral therapy for weight management and eating disorders in children and adolescents. Child Adolesc Psychiatr Clin N Am. 2011;20(2):271-85. Review.	ED and obesity in children and adolescents involve harmful behavior and attitude patterns that infiltrate daily functioning. CBT is well suited to treating these conditions, given the emphasis on breaking negative behavior cycles.This article reviews the current empirically supported treatments and the considerations for youth with weight control issues. New therapeutic modalities (*i.e*, enhanced CBT and the socioecologic model) are discussed.	The parallels between ED and obesity allow for the discussion of these issues along a weight control continuum. Within the ED field, specialized psychotherapies (*e.g*., CBT and IPT) remain effective modalities for the individual ED diagnoses, and a "transdiagnostic" approach (*i.e*., CBT-E) has been developed to better address symptom fluctuation between diagnostic. categories. For obesity, family-based behavioral treatment programs are the most effective, and the incorporation of targeted cognitive skills are useful additions. These lifestyle interventions are enhanced when applied through a socio-ecological framework. Across the spectrum, treatment approaches should encourage the family, peer network, and community to create supportive environments.	Rationale is provided for extending therapy beyond the individual treatment milieu to include the family, peer network, and community domains to promote behavior change, minimize relapse, and support healthy long-term behavior maintenance.

continue...

...Continuation

Extracted of PubMed

Table of findings - Eating disorders

Systematic review/meta-analysis	Objective/methods	Main results	Conclusions
Wanden-Berghe RG, Sanz-Valero J, Wanden-Berghe C. The application of mindfulness to eating disorders treatment: a systematic review. Eat Disord. 2011;19(1):34-48. Review.	The present study is an exploratory examination of the efficacy of the application of mindfulness-based interventions to the treatment of ED. Eight studies were included in the systematic review. Each study reported satisfactory results, although trial qualities were variable and sample sizes were small.	The current study found initial evidence supporting the effectiveness of mindfulness-based interventions to the treatment of ED.	The application of mindfulness-based interventions to the treatment of ED remains a promising approach worthy of further research.
Tirico PP, Stefano SC, Blay SL. Validity studies of quality of life instruments for eating disorders: systematic review of the literature. J Nerv Ment Dis. 2010;198(12):854-9.	To conduct a systematic review of studies that evaluated QOL among individuals with ED, to analyze the characteristics of specific QOL instruments for ED. Bibliographic searches were conducted in six databases and manual searches in two journals, covering the period from January 1975 to June 2008.	Forty-one studies met the inclusion criteria for this review, five reported on the validation process for 4 specific instruments for ED. Among the four specific QOL instruments for ED, three presented adequate development procedures and psychometric properties. However, further research is needed to prove the validity and applicability of these instruments.	Additional validation studies are needed, especially in relation to patients who deny that they have a disorder.
Menzel JE, Schaefer LM, Burke NL, Mayhew LL, Brannick MT, Thompson JK. Appearance-related teasing, body dissatisfaction, and disordered eating: A meta-analysis. Body Image. 2010;7(4):261-70. Review.	A meta-analysis was conducted to determine the relation between appearance and weight-based teasing and three outcome measures: body dissatisfaction, restrictive eating, and bulimic behaviors.	Four meta-analyses were conducted. Fifty effect sizes (n=10,618) resulted in a moderate effect size of.39 for the relation between weight teasing and body dissatisfaction; 24 effect sizes (n=7,190) resulted in an effect size of.32 for the relationship between appearance teasing and body dissatisfaction; 20 effect sizes (n=4,792) resulted in an effect size of 35 for the relationship between weight teasing and dietary restraint; and 22 effect sizes (n=5,091) resulted in an effect size of 36 for the relationship between weight teasing and bulimic behaviors. Significant moderators that emerged were teasing measure type, publication type, study type, age group, and sex.	The findings offer further support for the inclusion of strategies in body image and ED prevention and intervention programs that focus on handling negative, appearance-related commentary.
Varchol L, Cooper H. Psychotherapy approaches for adolescents with eating disorders. Curr Opin Pediatr. 2009;21(4):457-64. Review.	Adolescence is the most common period for the onset of ED, and early intervention is critical. Primary Care providers should feel equipped to discuss psychotherapy approaches for ED with adolescents and their families and to provide appropriate referrals. The present review focuses on six prominent treatment modalities and the evidence supporting each approach.	Although the majority of studies about psychotherapy approaches for ED focus on adult women, there is a growing body of research on effective treatments for an adolescent population. Family-based treatment (the Maudsley method) and supportive psychotherapy appear to be promising approaches for anorexia in teens. Treatments for bulimia yield extremely high relapse rates, but cognitive-behavioral therapy and family-based treatment are favored modalities. Dialectical behavior therapy and IPT may also be applicable to adolescent bulimia and binge eating. Most psychotherapists draw upon a variety of these treatment approaches, depending upon the patient's unique presentation. Regardless of the modality used, some degree of family involvement is important in limiting dropout and improving outcomes.	Adolescent health providers need to be aware of the psychotherapy approaches recommended for teens with ED in order to effectively refer patients to and collaborate with mental health providers.
Waxman SE. A systematic review of impulsivity in eating disorders. Eur Eat Disord Rev. 2009;17(6):408-25. Review.	To conduct a systematic review of the current literature that examines impulsivity in individuals with ED. Studies were obtained from Embase, PubMed e PsycINFO, and were included if they assessed impulsivity in individuals over 18 years of age with an ED diagnosis and published in the last 10 years. The methodological quality of the studies was rated. Twenty studies were included in this review, with methodological quality varying across studies.	Findings suggest that impulsivity is best assessed multi-modally, with a combination of self-report, behavioural and physiological measures. In general, impulsivity was found to differentiate individuals with ED from controls, as well as across diagnostic subtypes.	The current findings have important clinical implications for our understanding and treatment of both impulsivity and ED.

continue...

...Continuation

Extracted of PubMed

Table of findings - Eating disorders

Systematic review/meta-analysis	Objective/methods	Main results	Conclusions
Keel PK, Haedt A. Evidence-based psychosocial treatments for eating problems and eating disorders. J Clin Child Adolesc Psychol. 2008;37(1):39-61. Review.	This review provides a synthesis of existing data concerning the efficacy of various psychosocial interventions for ED in adolescent samples. Modes of therapy examined in adolescent samples include family therapy, cognitive therapy, behavioral therapy, and CBT mostly in patients with AN.	At this time, the evidence base is strongest for the Maudsley model of family therapy for AN. Evidence of efficacy for other treatments and other conditions is limited by several methodological factors including the small number of studies, failure to use appropriate control conditions or randomization procedures, and small sample sizes (*i.e.*, fewer than ten participants per treatment arm). Potential moderators and mediators of treatment effect are reviewed. Finally, results from adolescent studies are contrasted with those from adult studies of ED treatment.	Many studies of adult populations comprise late adolescent/young adult participants, suggesting that findings regarding the efficacy of CBT for BN in adults likely extend to older adolescent populations.
Roberts A, Cash TF, Feingold A, Johnson BT. Are black-white differences in females' body dissatisfaction decreasing? A meta-analytic review. J Consult Clin Psychol. 2006;74(6):1121-31. Review.	Proponents of the sociocultural model of ED have suggested that ethnic differences in body dissatisfaction may be diminishing as the thin ideal of beauty becomes more widely disseminated among minority women. In a meta-analysis, the authors examined temporal trends in Black-White differences and also examined whether these differences generalize across various age groups and measures.	Results confirmed more favorable body image evaluations among Black than White females, with the greatest differences at the age period of the early 20s. Although results confirmed that ethnic differences have diminished, this trend was limited to weight-focused measures. On more global body image measures, ethnic differences actually increased.	These results suggest that the relationship between Black-White ethnicity and body image is more complex than previously suggested.
Perkins SS, Murphy RR, Schmidt UU, Williams C. Self-help and guided self-help for eating disorders. Cochrane Database Syst Rev. 2006;(3):CD004191. Review.	Evaluate evidence from RCT/CCT for the efficacy of PSH/GSH with respect to ED symptoms, compared with waiting list or placebo/attention control, other psychological or pharmacological treatments (or combinations) in people with ED. A total of 20 RCT and 3 CCT were identified, all focusing on BN, BED, EDNOS or combinations of these, in adults, using manual-based PSH/GSH across various settings.	At end of treatment, PSH/GSH did not significantly differ from waiting list in abstinence from bingeing or purging, although these treatments produced greater improvement on other ED symptoms, psychiatric symptomatology and interpersonal functioning but not depression.Compared to other formal psychological therapies, PSH/GSH did not differ significantly at end of treatment or follow-up in improvement on bingeing and purging, other ED symptoms, level of interpersonal functioning or depression. There were no significant differences in treatment dropout.	PSH/GSH may have some utility as a first step in treatment and may have potential as an alternative to formal therapist-delivered psychological therapy. Future research should focus on producing large well-conducted studies of self-help treatments in ED including health economic evaluations, different types and modes of delivering self-help (*e.g*. computerised *versus* manual-based) and different populations and settings.
Johansson L, Ghaderi A, Andersson G. Stroop interference for food- and body-related words: a meta-analysis. Eat Behav. 2005;6(3):271-81. Review.	According to cognitive theories of ED, biased information processing in favour of dysfunctional attitudes about food and body appearance plays a vital role in the development and maintenance of such disorders. Data from 27 studies evaluating Stroop interference for food- and body-related words with negative overtones were included in a meta-analysis in order to investigate whether such processing biases are specific to eating disordered samples. Participants were females characterised as eating disordered, non-eating disordered but nevertheless over-concerned with body appearance and eating, and normal controls.	Mean Stroop interference for eating disordered females was of medium effect size (Cohen's d=0.48) and significantly larger than for both non-eating disordered females concerned with body appearance and eating, and normal control females (both d=0.21).	Stroop interference for eating disordered females was thus of fairly modest magnitude where it was unclear whether such interference is specific to this sample.

IIP: identity intervention programme; ED: eating disorder; AN: anorexia nervosa; BN: bulimia nervosa; SPI: supportive psychotherapy; BMI: body mass index; EDNOS: eating disorder not otherwise specified; DBT: dialectical behaviour therapy; SUD: substance use disorders. 95%CI: 95% confidence interval; EDE-Q: Eating Disorder Examination; RMT: readiness and motivation therapy; CBT: cognitive behavior therapy; SF-36: Medical Outcomes Study 36; CBT-E: enhanced cognitive behaviour therapy; BMI: body mass index; DSM-IV: 4th ed. of the Diagnostic and Statistical Manual of Mental Disorders; RCT: randomized controlled trials; GSH: guided self-help; ACS-90: Action Control Scale; TAS-26: 26-item Toronto Alexithymia Scale; CD: cognitive dissonance; NES: night eating syndrome; NESS: Night Eating Symptom Scale; HAES: Health-at-Every-Size; SD: standard deviation; BED: binge eating disorder; DSM-V: 5th ed. of the Diagnostic and Statistical Manual of Mental Disorders; FBT: family-based treatment; CBT-GSH: cognitive-behavioral therapy, guided-self-help; SSRI: selective serotonin reuptake inhibitors; 95%CI: 95% confidence interval; IPT: interpersonal psychotherapy; QOL: quality of life; CCT: controlled clinical trial; TCBM: mindfulness-based intervention.

Appendix 2. The main characteristics of the Cochrane Systematic Reviews

Population	Interventions	Comparison of interventions	Outcomes
1. Hay PPJ, Bacaltchuk J, Byrnes RT, Claudino AM, Ekmejian AA, Yong PY. Individual psychotherapy in the outpatient treatment of adults with anorexia nervosa. 2003. Assessed as up-todate: Feb 11, 2008			
Older adolescents and adults (aged >16 years) of any age or gender with AN (DSM-III, DSM-III-R, DSM-IV diagnostic criteria (APA 1994); ICD-10 (WHO 1992); Russell (1970).	Individual psychotherapies (time-limited), IPT, CAT, CBT.	The individual psychotherapies were compared to:<br>1. Usual treatment<br>2. Dietary advice<br>3. Waiting list.	Primary outcomes<br>Weight restoration to within the normal weight range (*e.g.* BMI)<br>Weight, mean BMI (weight in kg/height in metres, squared) at end of treatment.<br>Secondary outcomes<br>Recovery according to the Morgan 1975 narrow scale of:<br>1. A good outcome, namely normal body weight with normal menstruation or<br>2. Intermediate outcome, namely normal body weight with no menstruation<br>Mean eating disorder symptom scores<br>Proportion of study drop-outs or non-completers for any reason or any adverse event or experience<br>Patient satisfaction ratings<br>Level of side effects or negative effects of therapy<br>General psychiatric symptomatology<br>Level of depression<br>Level of interpersonal function.
2. Fisher CA, Hetrick SE, Rushford N. Family therapy for anorexia nervosa. 2010. Assessed as up-to-date: Jul 31, 2008			
Patients of any age or sex with a primary clinical diagnosis of AN either or both purging or restricting subtype based on DSM (APA 1994) or ICD criteria (WHO 1992) or clinicians' judgement, of any severity.	The main types of family therapy have been considered:<br>- Structured family therapy<br>- Systemic family therapy<br>- Strategic family therapy<br>- Family-based therapy and its variants<br>- Systemic behavioral family therapy<br>- Other types of therapies, including approaches using family therapy, but are less specific	The family therapy was compared to:<br>1. Standard treatment or usual<br>2. Educational interventions<br>3. Psychological interventions (family therapy or others types of psychological interventions)	Primary outcomes<br>1. Remission (by DSM or ICD or standardised scale measure for remission)<br>2. All cause mortality<br>Secondary outcomes<br>1. Relapse<br>2. Dropout<br>3. Family functioning<br>4. General functioning<br>5. Cognitive distortion<br>6. Weight
3. Hay PPJ, Bacaltchuk J, Stefano S, Kashyap P. Psychological treatments for bulimia nervosa and binging. 2009. Assessed as up-to-date: May 31, 2007			
Adults (aged >16 years) with bulimia nervosa, binge eating and/or EDNOS.	1. Cognitive behaviour psychotherapy or CBT: a psychotherapy that uses the specific techniques and model of CBT-BN, as described by Fairburn and colleagues (Fairburn 1993b), but not necessarily the number of sessions or specialist expertise. In trials of bulimia nervosa, data were analysed for both the broader "CBT" and the strict "CBT-BN".	The CBT was compared to:<br>1. No treatment, to include waiting list<br>2. Other psychotherapy approaches<br>- Nutritional counselling (as an adjunct to a psychological treatment)<br>- IPT<br>- Hypnotherapy<br>- Psychoanalytic or psychodynamic psychotherapy<br>- Any other psychotherapy including BWLT (for overweight binge eaters<br>- PSH	Primary outcomes<br>1. 100% abstinence from binge eating at the end of therapy<br>2. Mean bulimic symptom scores either from an eating disorders symptom rating scale, or the estimated binge frequency at end of therapy<br>Secondary outcomes<br>1. Side effects or negative effects of therapy<br>2. Proportion of non-completers due to any reason, and those due to adverse events<br>3. Mean scores at end of therapy on any scale measuring depressive symptoms.<br>4. General psychiatric symptomatology<br>5. Improvement in interpersonal functioning<br>6. Weight<br>7. Patient satisfaction by a validated questionnaire or interview schedule

continue...

...Continuation

Appendix 2. The main characteristics of the Cochrane Systematic Reviews

Population	Interventions	Comparison of interventions	Outcomes
4. Hay PPJ, Claudino AM, Kaio MH. Antidepressants versus psychological treatments and their combination for bulimia nervosa. 2001. Avaliado como atualizado: 12 de agosto de 2001. Assessed as up-to-date: Aug 12, 2001			
People with bulimia nervosa defined by clinical state description or diagnosed by Russell's (Russell 1979), DSM or ICD criteria. Participants with both purging and nonpurging type bulimia nervosa, as defined in DSM-IV (APA 1994), were included.	Antidepressants<br>CBT<br>Cognitive therapy<br>Behaviour therapy<br>Psychodynamic/psychoanalytic-oriented therapy<br>Interpersonal therapy<br>Supportive therapy<br>Nutritional counselling	The psychological treatments were compared to:<br>1. Antidepressants<br>2. Combination (psychological treatments plus antidepressants)	A. Efficacy<br>(i) The number of people per treatment group who did not show a remission in the bulimic symptoms, defined as 100% reduction in binge or purge episodes from baseline to endpoint<br>(ii) The number of people per treatment group who did not show a clinical improvement in the bulimic symptoms, defined as at least 50% reduction in binge or purge episodes from baseline to endpoint<br>(iii) The average difference in bulimic symptoms at endpoint<br>B. Comorbidity<br>(i) Average difference in the severity of depressive symptoms at the end of the trial<br>C. Acceptability of the treatment<br>(i) Number of people per treatment group dropping out during the trial for any cause.
5. Perkins SSJ, Murphy RRM, Schmidt UUS, Williams C. Self-help and guided self-help for eating disorders. 2006. Assessed as up-to-date: May 23, 2006			
People of any age, gender or chronicity with AN or bulimia nervosa or binge eating or EDNOS (DSM, ICD, Russell, 1979).	Manuals PSH - only materials and manuals GSH - materials more guide therapist.	The PSH and GSH were compared to:<br>1.Waiting list<br>2. Other formal psychological therapies<br>3. PSH *versus* GSH	Primary outcomes:<br>(a) Abstinence from bingeing<br>(b) Abstinence from purging<br>(c) Weight (BMI)<br>Secondary outcomes:<br>(a) Eating disorder symptomatology<br>(b) Weight restoration (BMI) to within normal range<br>(c) Proportion of non-completers or dropouts due to any reason, and those due to adverse events<br>(d) Patient satisfaction<br>(e) Adherence to self-help (*e.g.* percentage of material read; percentage of homework tasks completed)<br>(f) Side effects or negative effects of therapy<br>(g) Additional help seeking<br>(h) General psychiatric and mental state symptomatology<br>(i) Improvement in interpersonal functioning<br>(j) Mean scores on any scale measuring depressive symptoms<br>(k) Health care cost.

AN: anorexia nervosa; DSM: Diagnostic and Statistical Manual of Mental Disorders; ICD: International Classification of Diseases; IPT: interpersonal psychotherapy; CAT: cognitive analytic therapy; CBT: cognitive behavioural therapy; BMI: body mass index; EDNOS: eating disorders not otherwise specified; CBT-BN: cognitive behavioural therapy for bulimina nervosa; BWLT: behavioural weight loss treatment; PSH: pure self-help; GSH: guided self-help.

Appendix 3. Significant results of main outcomes of Cochrane Systematic Reviews

1. Family therapy *versus* usual treatment for anorexia nervosa, and family therapy *versus* psychological interventions for anorexia nervosa – remission after intervention

Outcome	Studies	Family therapy (n/N)	Usual treatment (n/N)	Relative effect (95%CI)
Remission	2 (Dare, 2001; Crisp, 1991)	20/42	5/39	RR: 3.83 (1.60-9.13)
**Outcome**	**Studies**	**Family Therapy (n/N)**	**Psychological interventions (n/N)**	**Relative effect (95%CI)**
Remission	4 (Ball, 2004; Dare, 2001; Robin, 1999; Russell, 1987 groups 1, 2 and 3)	36/76	29/73	RR: 1.13 (0.72-1.76)

Source: Fisher CA, Hetrick SE, Rushford N. Family therapy for anorexia nervosa. Cochrane Database Syst Rev. 2010;(4):CD004780. Review.

2. Antidepressants *versus* psychotherapy and their combination for bulimia nervosa – remission and dropouts

Outcome	Studies	Antidepressants (n/N)	Psychotherapy (n/N)	Relative effect (95%CI)
Remission	5 (Agras, 1992; Goldbloom, 1996; Leitenberg, 1994; Mitchell, 1990; Walsh, 1997)	99/124	69/113	RR: 1.26 (0.90-1.77)
Dropouts	4 (Agras, 1992; Goldbloom, 1996; Leitenberg, 1994; Mitchell, 1990)	39/96	15/88	RR: 2.18 (1.09-4.35)
**Outcome**	**Studies**	**Antidepressants (n/N)**	**Combination (n/N)**	**Relative effect (95%CI)**
Remission	4 (Agras, 1992; Leitenberg, 1994; Goldbloom, 1996; Walsh, 1997)	54/70	41/71	RR: 1.37 (0.98-1.91)
Dropouts	4 (Agras, 1992; Goldbloom, 1996; Leitenberg, 1994; Mitchell, 1990)	39/96	34/100	RR: 1.19 (0.69-2.05)
**Outcome**	**Studies**	**Psychotherapy (n/N)**	**Combination (n/N)**	**Relative effect (95%CI)**
Remission	6 (Agras, 1992; Fichter, 1991; Goldbloom, 1996; Leitenberg, 1994; Russell, 1995; Walsh, 1997)	84/132	64/125	RR: 1.21 (1.02-1.45)
Dropouts	6 (Agras, 1992; Fichter, 1991; Goldbloom, 1996; Leitenberg, 1994; Russell, 1995; Mitchell, 1990)	22/141	46/154	RR: 0.57 (0.38-0.88)

Source: Hay PP, Claudino AM, Kaio MH. Antidepressants versus psychological treatments and their combination for bulimia nervosa. Cochrane Database Syst Rev. 2001;(4):CD003385. Review.

3. Cognitive behavior therapy *versus* waiting list or no treatment for bulimia nervosa, binge eating (binging) and/or eating disorder not otherwise specified bulimic type, and Cognitive behavior therapy *versus* any other psychotherapy for bulimia nervosa, binge eating (binging) and/or eating disorder not otherwise specified bulimic type – remission and improvement in mean bulimic symptoms

Outcome	Studies	CBT (n/N)	Waiting list/No treatment (n/N)	Relative effect (95%CI)
Remission	8 (Agras, 1989; Griffiths, 1993; Lee, 1986; Telch, 1990; Wilfley, 1993; Gorin, 2003; Peterson, 1998; Treasure, 1996)	110/ 177	162/172	RR: 0.69 (0.61-0.79)
Mean bulimic symptoms	12 (Agras, 1989; Freeman, 1988; Griffiths, 1993; Lee, 1986; Leitenberg, 1988; Sundgot-Borgen, 2002; Telch, 1990; Wilfley, 1993; Wolf, 1992; Gorin, 2003; Peterson, 1998; Treasure, 1996)	240	225	SMD: -0.94 (-1.18- -0.70)
Mean depression scores	7 (Agras, 1989; Carter, 1998; Lee, 1986; Leitenberg, 1988; Telch, 1990; Wilfley, 1993; Gorin, 2003)	146	140	SMD: -0.69 (-1.08- -0,30)
**Outcome**	**Studies**	**CBT (n/N)**	**Other psychotherapy (n/N)**	**Relative effect (95%CI)**
Remission	10 (Agras, 2000; Cooper, 1995; Fairburn, 1991; Griffiths, 1993; Hsu, 2001; Walsh, 1997; Wilfley, 1993; Wilfley, 2002; Munsch, 2007; Nauta, 2000)	205/389	235/374	RR: 0.87 (0.74-1.02)
Mean bulimic symptoms	15 (Agras, 2000; Cooper, 1995; Fairburn, 1986; Fairburn, 1991; Freeman, 1988; Griffiths, 1993; Walsh, 1997; Wilfley, 1993; Wilfley, 2002; Kenardy, 2002; Garner, 1993; Agras, 1994; Munsch, 2007; Nauta, 2000; Porzelius, 1995)	480	461	SMD: -0.21 (-0.34- -0.09)

Source: Hay PP, Bacaltchuk J, Stefano S, Kashyap P. Psychological treatments for bulimia nervosa and binging. Cochrane Database Syst Rev. 2009;(4):CD000562. Review.
CBT: Cognitive behavior therapy; 95%CI: 95%confidence interval.

4. Pure self-help, guided self-help *versus* waiting list for eating disorders – improvement in eating disorder symptomatology, psychiatric symptoms and interpersonal functioning

Outcome	Studies	PSH/GSH (N)	Waiting list (N)	Relative effect (95%CI)
Remission	2 (Carter, 1998; Banasiak, 2005)	123	79	SMD: -0.71(-1.01- -0.41)
Psychiatric symptoms	2 (Banasiak, 2005; Carter, 1998)	123	79	SMD: -0.32(-0.51- -0.13)
Interpersonal functioning	2 (Carter, 2003; Banasiak, 2005)	110	84	SMD: -0.34(-0.67- -0.02)

Source: Perkins SS, Murphy RR, Schmidt UU, Williams C. Self-help and guided self-help for eating disorders. Cochrane Database Syst Rev. 2006;(3):CD004191. Review.
95%CI: 95% confidence interval; RR: relative risk; PSH: Pure self-help; GSH: guided self-help.
